# Non-Clozapine interventions in treatment-resistant schizophrenia: a systematic review and meta-analysis

**DOI:** 10.1038/s41380-025-03255-y

**Published:** 2025-10-03

**Authors:** Richard Carr, Alistair Cannon, Valeria Finelli, Bernard Bukala, Yesim Cimen, Patritsiya Filipova, Connor Cummings, Toby Pillinger, Oliver D. Howes, Robert A. McCutcheon

**Affiliations:** 1 Department of Psychosis Studies, Institute of Psychiatry, King’s College London, London, UK; 2 South London & the Maudsley NHS Trust, London, UK; 3 London Institute of Medical Sciences, London, UK; 4 Department of Psychiatry, University of Oxford, Oxford, UK; 5 Oxford Health NHS Foundation Trust, Oxford, UK

**Keywords:** Schizophrenia, Psychiatric disorders

## Abstract

**Background and hypothesis:**

Clozapine is the only licensed pharmacotherapy for treatment resistant schizophrenia (TRS), but in some cases is not a suitable treatment option. A review of the efficacy of non-clozapine interventions in TRS may help inform clinical decision making when clozapine treatment is not feasible.

**Study design:**

A systematic review and meta-analysis was performed investigating the efficacy of non-clozapine augmentation of antipsychotic treatment in TRS on positive, negative, and total symptoms. The review protocol is registered at PROSPERO (ID: CRD42023418053). PsycInfo, PubMed and EMBASE were searched up until July 2023. Cochrane Risk of Bias tool (v2) was used to assess study quality. Data were pooled using a random-effects model for each class of intervention to give an estimate of effect size (Hedges’ *g*).

**Results:**

78 studies were included, of which 68 were included in the meta-analysis, comprising 3241 patients. High-dose antipsychotics (7 studies, 467 participants) did not improve any symptom domain. Augmentation of antipsychotics with glycine modulatory site agonists (9 studies, 187 participants) improved positive (g = −0.56 [−0.81, −0.31], GRADE rating Low), negative (g = −1.18 [−1.49, −0.87], GRADE rating Low) and total (g = −1.17 [−1.75, −0.59], GRADE rating Very Low) symptoms. Non-invasive stimulation (26 studies, 893 participants) moderately benefited positive symptoms (g = −0.42 [−0.65, −0.18], GRADE rating Low). Psychotherapy (10 studies, 565 participants) moderately improved positive symptoms (g = −0.56 [−1.01, −0.10], GRADE rating Low). Augmentation with antidepressants (3 studies, 187 participants) improved negative (g = −0.74 [−1.46, −0.02], GRADE rating Very Low) and total (g = −0.69 [−1.00, −0.38], GRADE rating Low) symptoms. Sample sizes were small, and publication bias was apparent for non-invasive stimulation studies.

**Conclusions:**

Several augmentation strategies, including pharmacotherapy, non-invasive stimulation, and psychotherapy demonstrated benefit in small studies, however no intervention reached the threshold of evidence to be routinely recommended as a viable alternative to clozapine. High-quality trials are needed for definitive recommendations.

## Introduction

Treatment resistant schizophrenia (TRS) is defined as schizophrenia that fails to respond to at least two separate adequate trials of non-clozapine antipsychotics at therapeutic dose, despite good adherence [1]. At 10-year follow-up from first presentation, around 33% of people with schizophrenia met criteria for treatment-resistance [2]. Current guidelines recommend that TRS is promptly treated with clozapine [3]; early commencement of which improves outcomes significantly [4]. Some patients, however, are unable to tolerate or otherwise unwilling to consider treatment with clozapine due to its side effect burden and monitoring requirements, while it is contraindicated in others [5].

Neither the neurobiological basis of treatment resistance, nor the pharmacological profile underlying clozapine’s superior efficacy are well understood [6, 7]. This contributes to the fact that there are no clearly established alternatives to clozapine. Despite this lack of a clear evidence base, a frequent problem facing the clinician is what intervention to offer the patient with refractory symptoms when alternatives to clozapine are needed. In view of this, while clozapine is first-line treatment in people with TRS, we conducted a systematic review and meta-analysis of the evidence for non-clozapine interventions to treat positive, negative and total psychopathology in people with treatment resistant schizophrenia.

## Methods

The protocol for the review and meta-analysis was preregistered at PROSPERO (ID: CRD42023418053). This work was not funded by any external sponsor.

### Search strategy

PsycINFO, EMBASE, ClinicalTrials.gov and PubMed were searched with the following terms up to July 2023:

((schizophrenia OR schizoaffective OR psychosis OR psychotic) AND (resistan* OR refractory*) AND (randomised control* trial OR randomized control* trial OR RCT OR double blind* OR double-blind* OR meta-analysis OR meta analysis OR Cochrane OR guidelines))

For the PubMed search, the following search indicators were also included:

ti, ab, kw

No restrictions on date were included.

### Scope of included studies

We aimed to include studies investigating the change in psychiatric rating scales for positive, negative and total symptoms following augmentation of non-clozapine antipsychotics with pharmacological, psychotherapeutic, nutritional, or neurostimulatory interventions, in patients with documented inadequate response to at least one trial of antipsychotics.

### Criteria for study selection

Studies included in the analysis were:

1. Randomised controlled trials with a suitable comparator (placebo or other control intervention, but not treatment as usual (TAU)).
2. Investigating therapeutic interventions in adult patients with treatment-resistant schizophrenia or schizoaffective disorder, defined as explicit inadequate response to at least one trial of antipsychotic medication.
3. Reporting baseline and endpoint scores on psychiatric rating scales assessing positive, negative and total symptoms.

Studies were excluded if they were:

1. Trials explicitly investigating augmentation of clozapine.
2. Trials investigating dopamine receptor antagonists compared to placebo treatment (see Supplemental Methods for rationale).
3. Trials reporting primary endpoints not related to psychopathology (e.g., investigating treatments to manage cardiometabolic risk).

While treatment-resistance is currently defined according to the Treatment Resistance and Response in Psychosis (TRRIP) consensus criteria, we also included studies with a more lenient definition of treatment resistance, namely at least one documented failed trial of antipsychotics. We did this to capture more studies in the analysis, and as many of the included studies were published before the TRRIP consensus was reached. We later conduct post-hoc tests to examine the effect of including these studies.

While explicit clozapine augmentation studies were excluded, most studies, where this information was reported, included some patients on clozapine in the sample. These studies were included, given the fact that studies which did not report specific antipsychotics may have also included such patients. The effect of including clozapine patients was assessed using meta-regression.

We excluded studies using TAU as a comparator. This was most commonly seen in psychotherapy trials. We chose to do this since in TAU, participants are aware they are not receiving a study intervention, and as such can show a nocebo effect. The intensity of TAU can vary widely between studies, with less-intense forms showing similar effect sizes to waitlist control [8], a similar form of inactive control, where there is effectively no placebo condition meaning effect sizes can be greatly inflated [9].

### Paper screening, data synthesis and quality assessment

Papers identified using the search strategy were screened twice independently (AC, CC, YC, PF, then RC and BB). Any discrepancies were escalated to RM who adjudicated on inclusion. Data were then extracted twice independently (AC, CC, RC, VF). Discrepancies were again reviewed and adjudicated by RM.

Data extracted included: study author, date, TRS definition, intervention and comparator, rating scales used, level of blinding, baseline medication use, sample size, baseline symptom severity scale scores in each group and standard deviation (SD), mean and SD of the change in rating scale score, mean age and gender distribution of each group, and which antipsychotics and dose chlorpromazine equivalents (CPZE) subjects were taking at trial entry. Where results from multiple rating scales were reported, we extracted the Positive and Negative Symptom Scale (PANSS), except in non-invasive stimulation (NIS) studies. Since these were often explicitly focused on alleviating auditory hallucinations, hallucination-specific rating scales were extracted instead for positive symptoms. In most cases CPZE doses were given in subject demographics tables, but in some cases CPZE doses were estimated from doses of other antipsychotics (if reported) using the minimum effective dose method [10]. WebPlotDigitizer (version 4.6) [11] was used to extract data from graphs. Where change scores and SD were not reported, these were imputed from baseline and follow-up scores (see Supplement). If multiple time points were reported, data from the timepoint closest to 12 weeks was extracted, with the exception of non-invasive stimulation studies, which were extracted at end-of-treatment as these were typically of much shorter duration, and not all reported longer-term follow-up assessments. Where studies reported change scores as a percentage of baseline plus SD, rather than as an absolute value, these were converted to absolute values using the baseline score. Cochrane Risk of Bias (RoB) [12] tool v2 was used to assess for study quality. Three authors (RC, BB, VF) assessed study quality, giving a RoB score of low, medium, or high. These scores were visualised using the *robvis* tool [13].

### Data analysis

R (version 4.2.2) [14], with the metafor package (version 3.8-1) [15], was used for analysis by RC. Where pharmacological interventions fit into a broadly accepted therapeutic class (e.g., antipsychotic, antidepressant), we used these classes. Where interventions did not fit into a therapeutic class (e.g., glycine, d-cycloserine, d-serine) we classified them by their pharmacological action. Where there were >1 studies investigating a specific drug that did not fit into these therapeutic or pharmacological classes (e.g. sodium nitroprusside, ondansetron, famotidine and *Ginkgo biloba*), they were analysed individually. Interventions were thus classified as belonging to the following classes: mood stabiliser, antidepressant, antipsychotic, glycine modulatory site agonist (including full- and partial-agonist subgroups), NIS (including repetitive transcranial magnetic stimulation (rTMS) and transcranial direct current stimulation (tDCS) as subgroups), psychotherapy, ondansetron, famotidine, sodium nitroprusside, *Ginkgo biloba*. Effect size, presented as standardised mean difference (SMD, or Hedges’ *g*) of change scores, was calculated for each study. Effect sizes were then combined in a random effects meta-analysis for each class of intervention. Heterogeneity was estimated using I^2^. Meta-regressions were planned to investigate the effects of antipsychotic dose, the inclusion of clozapine patients, and baseline symptom severity on SMD. Wald-type tests were planned to investigate whether variables such as the definition of TRS used by studies and risk of bias were significant predictors of effect size. Publication bias was investigated using funnel plots, where there were ≥8 studies in a given intervention class, and Egger’s tests, where there were ≥10 studies [16]. Sensitivity analyses were performed to investigate robustness of results where post-hoc tests suggested that study factors, e.g. the definition of TRS used, influenced effect size. Grading of Recommendations, Assessment, Development and Evaluation (GRADE) assessment was used to assess certainty in the outcomes. Ratings are presented with each outcome reported.

## Results

### Systematic search results

**Fig. 1:**
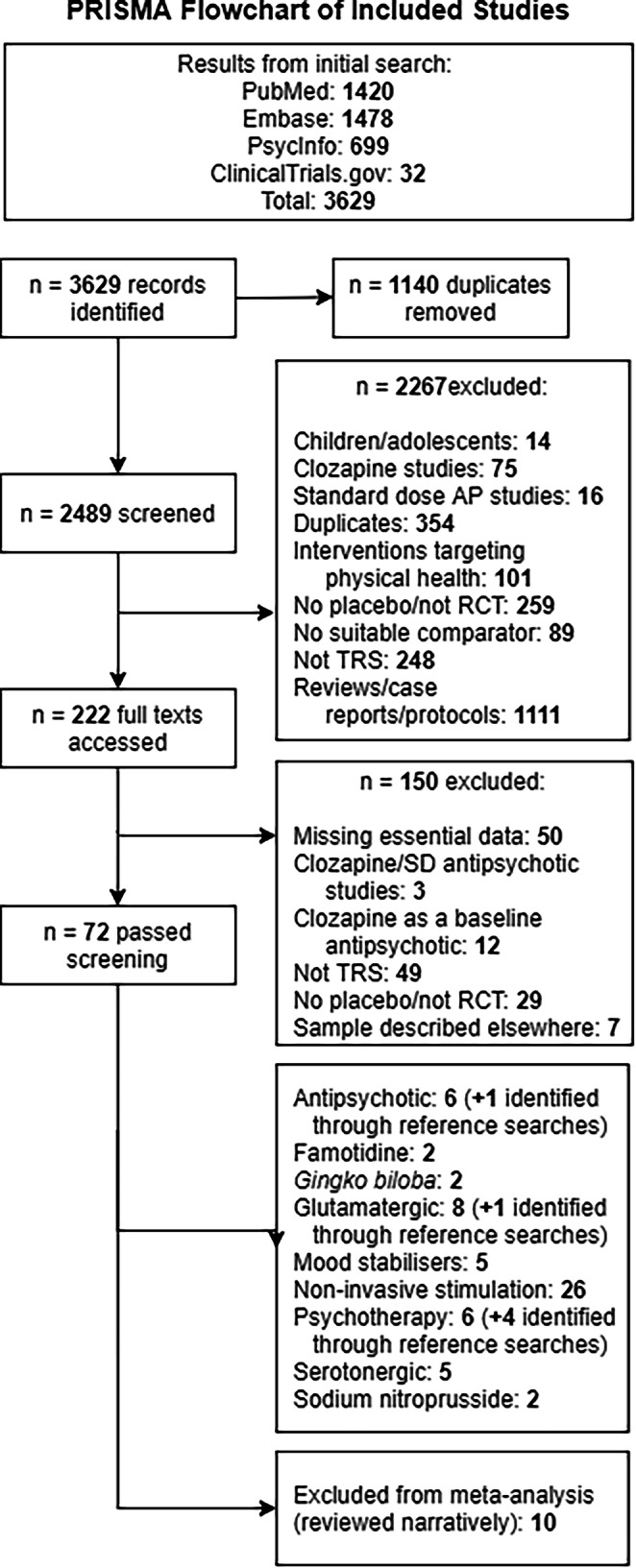
PRISMA flowchart of the studies included in the systematic review. AP antipsychotic, RCT randomised controlled trial, SD standard dose, TRS treatment resistant schizophrenia.

The Preferred Reporting Items for Systematic Reviews and Meta-Analyses (PRISMA) flowchart is presented in Fig. 1. The search, performed in June 2024, identified 3629 potential studies. 1140 duplicates were removed. 2267 papers were screened out on abstract review, leaving 222 papers which were accessed for full-text screening, of which 72 studies passed screening. An additional six studies [17–22] were found through screening reference lists in relevant review articles that were identified in the search. The resulting studies are summarised in Table 1. 68 of these, comprising 3241 patients, were included in the meta-analysis. A remaining 10 studies examined interventions not fitting into a clear specific intervention class, and so were not included in the meta-analysis but were reviewed narratively. While two of these investigated allopurinol [23, 24], as baseline symptom scores were missing from one [24], they were not included in the meta-analysis.

**Table 1:** Studies included in the systematic review.

Authors & Country	Level of Blinding	TRS definition	Intervention	Sample size			Outcome measure(s)	Risk of bias
				I		C		
Adelino et al., [62], Brazil	Double	Nonresponse to 2+ trials of APM for >6w	Sodium nitroprusside infusions, 0.5 mcg/kg/min, every 2 weeks	10		10	PANSS	Some
Bais et al., [74], Netherlands	Double	Daily AVH despite 2+ trials of APM given for >4w	1 Hz rTMS BD to left TPJ for 20 m, or to the left TPJ for 10 m and then the right TPJ for 10 m	16 (left), 15 (right)		16	AHRS, PANSS	Some
Bjørndal et al., [41], Denmark	Double	Unsatisfactory antipsychotic effect of previous neuroleptic treatment, duration unspecified	Haloperidol 10–240 mg/day	12		11	BPRS	High
Blumberger et al., [75], Canada	Double	Daily AVH despite 2+ trials of APM for >6w	1 Hz rTMS to left Heschl’s gyrus for 20 m daily, or 1 Hz rTMS to left Hesch’s gyrus for 10 m preceeded by 10 Hz priming for 10 m	17 (priming), 17 (no priming)		17	PSYRATS, PANSS	Low
Bose et al., [76], India	Double	Persistent AVH despite 1+ trial of APM given for >3 m	2 mA tDCS between left PFC and left TPJ, BD for 20 m	12		12	AHRS	Low
Brown et al., [63], USA	Double	No clinically significant reduction in symptoms following 1+ trial of APM for >8w	Sodium nitroprusside, 0.5ug/kg/min over 4 h, 1 or 2 doses	37		23	PANSS	Some
Brunelin et al., [78], France	Double	“Medication-resistant AVH”, unspecified	1 Hz rTMS to left T3P3, 1000 stimulations per session, BD for 5 consecutive weekdays	14		10	AHRS	High
Brunelin et al., [77], France	Double	Daily AVH refractory despite 1+ adequate trial of APM for >3 m	2 mA tDCS between left PFC and left TPC, 20 m BD for 5 consecutive weekdays	15		15	AHRS, PANSS	Low
Brunstein et al., [23], Brazil	Double	Persistent psychotic symptoms despite 2+ trials of APM for >6w	Allopurinol 300 mg BD for 6 weeks, **crossover**	23		23	PANSS	High
Cather et al., [19], USA	Single (raters blinded)	Persistent psychotic symptoms despite treatment with olanzapine for >6 m	Functional CBT, weekly sessionf ro 16w, compared to psychoeducation	15		13	PSYRATS, PANSS	Some
Chang et al., [80], Taiwan	Double	Persistent AVH despite 1+ trial of APM for >3 m	2 mA tDCS between left FPC and left TPJ, 20 m BD for 5 consecutive weekdays	30		30	AHRS, PANSS	Low
Chauhan et al., [95], India	Double	No period of good social or occupational functioning despite 2+ trials of APM given for >6w	5–50 Hz iTBS to left cerebellum, 600 pulses per session, BD for 5 consecutive weekdays	16		14	PANSS	High
Ding et al., [57], China	Double	“Treatment-resistant schizophrenia”, unspecified	Escitalopram, up to 20 mg OD for 8w	28		26	PANSS	High
Durham et al., [39], Scotland	Single (raters blinded)	Persistent and distressing positive symptoms despite stable dose of APM for 6 m	Cognitive behavioural therapy, 20 sessions over 9 m	22		23	PANSS	High
Farzin et al., [64], Iran	Double	No response to 2+ courses of APM	Famotidine 60 mg OD for 6w	15		15	PANSS	Some
Fitzgerald et al., [81], Australia	Double	No response to 2+ trials of APM given for >8 weeks	1 Hz rTMS to left TPJ, 15 m OD for 10 consecutive weekdays	17		16	PSYRATS	Some
Fitzgerald et al., [82], Australia	Double	No response to 2+ adequate trials of APM given for >8w	10 Hz rTMS to bilateral PFC, 25 m OD for 15 consecutive weekdays	12		8	PANSS, SANS	Some
Fröhlich et al., [79], USA	Double	Ongoing auditory hallucinations despite 2+ adequate trials of APM, duration unspecified	2 mA tDCS between left FPC and T3P3, 20 m OD for 5 days	13		13	AHRS, PANSS	Low
Goff et al., [55], USA^a^	Double	Significant positive symptoms despite APM for at least 4w	Lamotrigine 100–400 mg OD for 4w	104		105	PANSS, SANS	Low
Goff et al., [55], USA^a^	Double	Significant positive symptoms despite APM for at least 3 m	Lamotrigine 100–400 mg OD for 4w	104		105	PANSS, SANS	Low
Gornerova et al., [93], Czech Republic	Double	AVH refreactory 2+ adequate trials of APM, duration unspecified	0.9 Hz rTMS to left TPJ, 22 m OD for 10 consecutive weekdays	10		9	PANSS	Low
Goswami et al., [72], India	Double	Nonresponse to 3+ adequate trials of APM given for >6w	Electroconvulsive therapy, 3 sessions per week for 4w	15		10	BPRS, CGI	Some
Haddock et al., [20], UK	Single (raters blinded)	Persistent delusions or hallucinations despite treatment with adequate dose of APM, duration unspecified	CBT, 25 sessions over 6 months	39		38	PANSS	Some
Heresco-Levy et al., [45], Israel^b^	Double	Nonresponse to 3+ adequate trials of APM from 2 different classes given for >6w	20% glycine solution up to 0.8 g/kg/day for 6w, **crossover**	12		12	PANSS	Some
Heresco-Levy et al., [47], Israel^b^	Double	Nonresponse to 3+ adequate trials of APM from 2 different classes given for >6w	D-cycloserine, 25 mg BD for 6w, **crossover**	9		9	PANSS	Low
Heresco-Levy et al., [46], Israel^b^	Double	Nonresponse to 3+ adequate trials of APM from 2 different classes given for >6w	20% glycine solution, up to 0.8 g/kg/day for 6w, **crossover**	19		19	PANSS	High
Heresco-Levy et al., [25], Israel^b^	Double	Nonresponse to 3+ adequate trials of APM from 2 different classes given for >8w	D-cycloserine, 60 mg OD for 6w, **crossover**	16		16	PANSS	High
Heresco-Levy et al., [27], Israel^b^	Double	Nonresponse to 3+ adequate trials of APM from 2 different classes given for >8w	20% glycine solution up to 0.8 g/kg/day for 6w, **crossover**	17		17	PANSS	Low
Heresco-Levy et al., [26], Israel^b^	Double	Nonresponse to 3+ adequate trials of APM from 2 different classes for >8w	D-serine, 30 mg/kg/day for 6w, **crossover**	18		18	PANSS, SANS, BPRS	Low
Hoffman et al., [83], USA	Double	AVH refractory to APM given for >6 months	1 Hz rTMS to left T3P3 4, 8, 12, and 16 m sessions OD	6		6	HCS	Low
Hoffman et al., [94], USA	Double	AVH refractory to 2+ adequate trials of APM given for >6w	1 Hz rTMS ro left T3P3, 16 m OD for 9 consecutive weekdays, **crossover**	12		12	HCS	Low
Javitt et al., [110], USA^b^	Double	Nonresponse to adequate trials of typical and atypical APM given for >6 m	20% glycine solution, up to 0.8 g/kg/day	12		12	PANSS	Low
Kantrowitz et al., [84], USA	Double	Significant AVH despite treatment with APM for >4w	2 mA tDCS between left PFC and left TPJ, 20 m BD for 5 consecutive weekdays	47		42	AHRS, PANSS	Low
Karpouzian-Rogers et al, [44], USA^a^.	Not stated	Failure to respond to 2+ trials of APM given for >6w	Lurasidone 240 mg OD given for 24w	22		21	PANSS	High
Kimura et al., [28], Japan	Double	AVH refractory to 2+ adequate trials of APM given for >4w	20 Hz rTMS to left T3P3, BD for 2 consecutive days	16		14	AHRS	Low
Kinon et al., [17], USA	Double	Non-response to fluphenazine given for 4w	Fluphenazine 80 mg OD for 4w	18		16	BPRS, SANS	High
Klirova et al., [85], Poland	Double	“Medication-resistant AVH”, unspecified	0.9 Hz rTMS to left T3P3, 10 sessions	10, 10 (T3P3, NN)		10	PANSS	High
Koops et al., [86], Netherlands	Double	Insufficient treatment response to 2+ adequate trials of APM, duration unspecified	2 mA tDCS between left dlPFC and left TPJ, 20 m BD for 5 consecutive days	26		18	AHRS	Some
Kremer et al., [54], Israel	Double	Unsatisfactory response to 2 + APM for >8w	Lamotrigine, up to 400 mg OD for 10w	25		13	PANSS, SANS, BPRS	Some
Kulkarni et al., [66], Australia	Double	Persistent positive symptoms despite treatment with APM for >4w	Oestradiol 100–200 mcg transdermal every 3.5 d for 8w	59, 62 (100, 200 mcg)		62	PANSS	Low
Lee et al., [87], South Korea	Double	Nonresponse to 2+ adequate trials of APM given for >4w	1 Hz rTMS between T3/4 and P3/4, 20 m OD for 10 consecutive days	13, 12 (left, right)		14	AHRS	Low
Lindenmayer et al., [30], USA	Double	Positive symptoms refractory to 1+ adequate trials of APM given for >6w, plus nonresponse to quetiapine at standard dose	High-dose quetiapine, up to 1200 mg OD for 8w	29		31	PANSS	Low
Lindenmayer et al., [29], USA	Double	Nonresponse to 2+ adequate trials of APM ‘of adequate duration’	2 mA tDCS between left FC and left TPJ, 20 m BD for 5 consecutive days	15		13	PANSS	Low
Marco et al., [68], USA^c^	Double	Residual symptoms despite treatment with APM for >6w	Ketoconazole, up to 800 mg OD for 4w	7		8	HDRS	Low
McIntosh et al., [88], Scotland	Double	AVH refractory to APM for at least 2 months	1 Hz rTMS, 4, 8, 12, and 16 m sessions to left T3P3 over 4 d	16		16	PANSS	Low
Mellin et al., [89], USA^d^	Double	AVH refractory to 2+ trials of APM, duration unspecified	2 mA tDCS or tACS between F3/FP1 to T3P3, BD for 5 consecutive days	8, 7 (tACS, tDCS)		7	AHRS, PANSS	High
Meltzer et al., [31], USA	Double	Persistent psychotic symptoms despite 2+ trials of APM, duration unspecified	Risperidone, 100 mg biweekly for 24w	78		82	PANSS	High
Meltzer et al., [43], USA^a^	Double	Failure to respond to 2+ trials of APM given for >6w	Lurasidone, 240 mg OD for 24w	22		22	PANSS	High
Mishra et al., [67], India	Double	Nonresponse to 2+ adequate trials of APM given for >6w	Alpha-lipoic acid, 300 mg OD for 8w	10		10	SAPS, SANS	Some
Miyaoka et al., [71], Japan	Double	Little or no response to 2+ adequate trials of APM given for >4w	TJ-54 (yokukansan) 2.5 g TDS for 4w	50		48	PANSS	Low
Modabber et al., [24], Pakistan	Double	Symptoms refractory to 2+ trials of APM given for >6 m	Allopurinol 300 mg BD for 6 m, **crossover**	17		17	PANSS	Low
Mondino et al., [96], France	Double	Daily AVH despite treatment with APM for >3 m	2 mA tDCS between F3/FP1 and T3/P3, 20 m BD for 5 consecutive days	12		11	PANSS, AHRS	Low
Paillére-Martinot et al., [90], France	Double	AVH refractory to ≥2 trials of APM given for >4w	1 Hz rTMS to TPJ, 20 m OD for 10 consecutive days	15		13	SAPS, SANS	Some
Penn et al., [21], USA	Single (raters blinded)	Persistent AVH despite 2+ trials of APM for >8w	CBT, 12 sessions over 3 months	32		33	PANSS	Some
Peselow et al., [69], USA	Double	Active positive symptoms despite treatment with APM for >3w	CCK-8 0.04–0.08 mcg/kg, single dose, crossover	27		27	BPRS	Some
Poulet et al., [91], France	Double	AVH refractory to 2+ trials of APM given for >6w	0.9 Hz rTMS to left T3P3, 16 min BD for 5 consecutive weekdays	10		10	AHRS	Low
Rahmanzadeh et al., [73], Iran	Double	AVH refractory to 2+ adequate trials of APM given for >6w	Bumetanide, 1 mg BD for 8w	12		12	PANSS	High
Sakurai et al., [42], Japan	Double	Persistent symptoms despite treatment with APM for >4w	Olanzapine 20 mg or risperidone 6 mg for 4w	52		51	PANSS	High
Samadi et al., [59], Iran	Double	Persistent symptoms despite treatment with APM for >2 m	Ondansetron, 4–8 mg OD for 12w	18		20	PANSS	High
Sensky et al., [36], UK	Single (raters blinded)	Significant symptoms despite adequate trials of APM for >2w	Cognitive behavioural therapy, once weekly for 8w, compared to befriending	46		44	CPRS, SANS	Low
Shawyer et al., [37], Australia	Single (raters blinded)	Command hallucinations despite treatment with APM, duration unspecified	Acceptance and commitment therapy, 15 sessions given weekly, compared to befriending	17		18	PANSS	Some
Shawyer et al., [35], Australia	Single (raters blinded)	Persistent positive symptoms despite treatment with APM for >6 m	Acceptance and commitment therapy, 8 sessions weekly to fortnightly, compared to befriending	49		47	PANSS	Low
Sheitman et al., [70], USA	Double	Severe residual symptoms despite 3+ adequate trials of APM, duration unspecified	Intravenous secretin, 75 clinical units, single dose	11		11	PANSS	Low
Shi et al., [34], China	Double	Nonresponse to at 2+ trials of APM given for >6w	Sertraline 50 mg OD for 24w	53		62	PANSS	Low
Shiloh et al., [32], Israel	Double	Noneresponse to 3+ adequate trials of APM given for >6w	Mianserin, 30 mg OD for 6w	9		9	SAPS, SANS, BPRS	Low
Slotema et al., [92], Netherlands	Double	AVH refractory to 2+ trials of APM given for >6w	1 Hz rTMS, to left T3P3 or neuronavigated, 20 m OD for 15 sessions	22, 20 (T3P3, NN)		20	PANSS	High
Tarrier et al., [18], UK	Unblinded	Psychotic symptoms not responding to APM given for >6 m	CBT (coping strategy enhancement), weekly for 5w, compared to problem solving sessions	15		12	BPRS (Psychotic symptoms)	High
Tiihonen et al., [53], Sweden	Double	Nonresponse to atypical APM given for >4 m	Topiramate, up to 300 mg/day in addition to regular APM for 12w, **crossover**	23		23	PANSS	Low
Tsai et al., [49], USA	Double	Nonresponse to 2+ trials of APM given for >4w	D-serine, up to 30 mg/kg daily for 6w	14		15	PANSS, SANS, CGI	Low
Tyagi et al., [97], India	Single (raters blinded)	Inadequate response to APM given for >6w	50 Hz cTBS, between T3P3 and T4P4, BD for 10 consecutive weekdays	30		29	AHRS, PANSS	High
Valmaggia et al., [40], Netherlands	Single (raters blinded)	Residual AVH or delusions for at least 3 months, despite APM for >6w	16 h of CBT over 22 weeks, compared to supportive counselling	35		23	PANSS	High
Van Bercken et al., [22], Netherlands	Double	Moderate-severe symptoms despite treatment with APM for >6 m	D-cycloserine, 50 mg BD for 8w	13		13	PANSS	Low
Wilson et al., [56], USA^c^	Double	Persistent psychosis despite treatment with APM for >8w	Lithium carbonate, dose titrated, OD for 8w.	11		10	BPRS, SANS	Low
Xue et al., [38], China	Single (raters blinded)	Persistent symptoms despite ≥2 adequate trials of APM, duration unspecified	Meditation, 30 mins daily for 8 months		32	32	PANSS	Low
Zhang et al., [60], USA	Double	Nonresponse to ≥2 adequate trials of APM given for >3 m	*Ginkgo biloba* extract, 360 mg/day, in addition to normal APM for 12w		56	53	SAPS, SANS, BPRS	Low
Zhang et al., [58], China	Double	Nonresponse to ≥2 adequate trials of APM for >6w	Ondansetron 8 mg/day for 12w		58	63	PANSS	Low
Zhou et al., [61], China	Double	Ongoing symptoms despite 2+ trials of APM given for >6 m	*Gingko biloba* extract, 360 mg OD for 12w		27	27	SAPS, SANS	High

*APM* antipsychotic medication, *AHRS* auditory hallucinations rating scale, *AVH* audioverbal hallucinations, *BD* twice daily, *BPRS* brief psychiatric rating scale, *CBT* cognitive behavioural therapy, *CGI* clinical global impression, *CPRS* comprehensive psychopathology rating scale, *DSM* diagnostic and statistical manual, *HCS* hallucination change scale, *ICD* international classification of diseases, *NN* neuronavigated, *OD* once daily, *PANSS* positive and negative symptom scale, *PFC* prefrontal cortex, *PSYRATS* psychotic symptom rating scale, *rTMS* repetitive transcranial magnetic stimulation, *SANS* scale for assessment of negative symptoms, *SAPS* scale for assessment of positive symptoms, *tA/DCS* transcranial alternating/direct current stimulation, *TDS* three times daily, *TPJ* temporo-parietal junction, *TRS* treatment resistant schizophrenia.

^a^Study was funded by study drug manufacturer.

^b^One of the study authors acquired the intellectual property rights to the glycine modulatory site agonist d-serine in 2000.

^c^Study drug was provided free of charge by pharmaceutical companies.

^d^One author has founded a company marketing noninvasive stimulation devices.

### Characteristics of included studies and participants

All studies included patients on baseline non-clozapine antipsychotic therapy, typically continuation of the participant’s current treatment. Although studies specifically investigating clozapine augmentation were excluded, many studies included patients on clozapine in their sample. The proportion of patients on clozapine was reported in 44 studies included in the meta-analysis, with a mean proportion (excluding studies where no patients took clozapine) of 31% [25–34].

Most studies used placebo or sham treatment as a comparator, however psychotherapy studies used active control. Active controls included interventions such as psychoeducation [19], social activities [20], befriending [35–37], general rehabilitation [38], problem-solving [18] and supportive counselling [21, 39, 40]. High-dose antipsychotic studies compared against lower doses of the same antipsychotic.

Median total sample size was 29.5. Where recorded, participants had a mean age of 41.3 years and a mean baseline antipsychotic dose of 632.6 mg CPZE. 63.1% of participants were male. 29 studies included participants with schizoaffective disorder, with a mean proportion of 26%. The majority of studies used a definition similar to the TRRIP consensus, i.e., inadequate response to at least two adequate trials of antipsychotics (Table 1), and several included criteria for dose and duration. 32 studies used a less conservative definition, requiring only one period of non-response. Where no precise definition was specified, this was assumed to be the least conservative, so long as it was still explicit that some period of non-response was required for inclusion.

### Pharmacological interventions

**Fig. 2:**
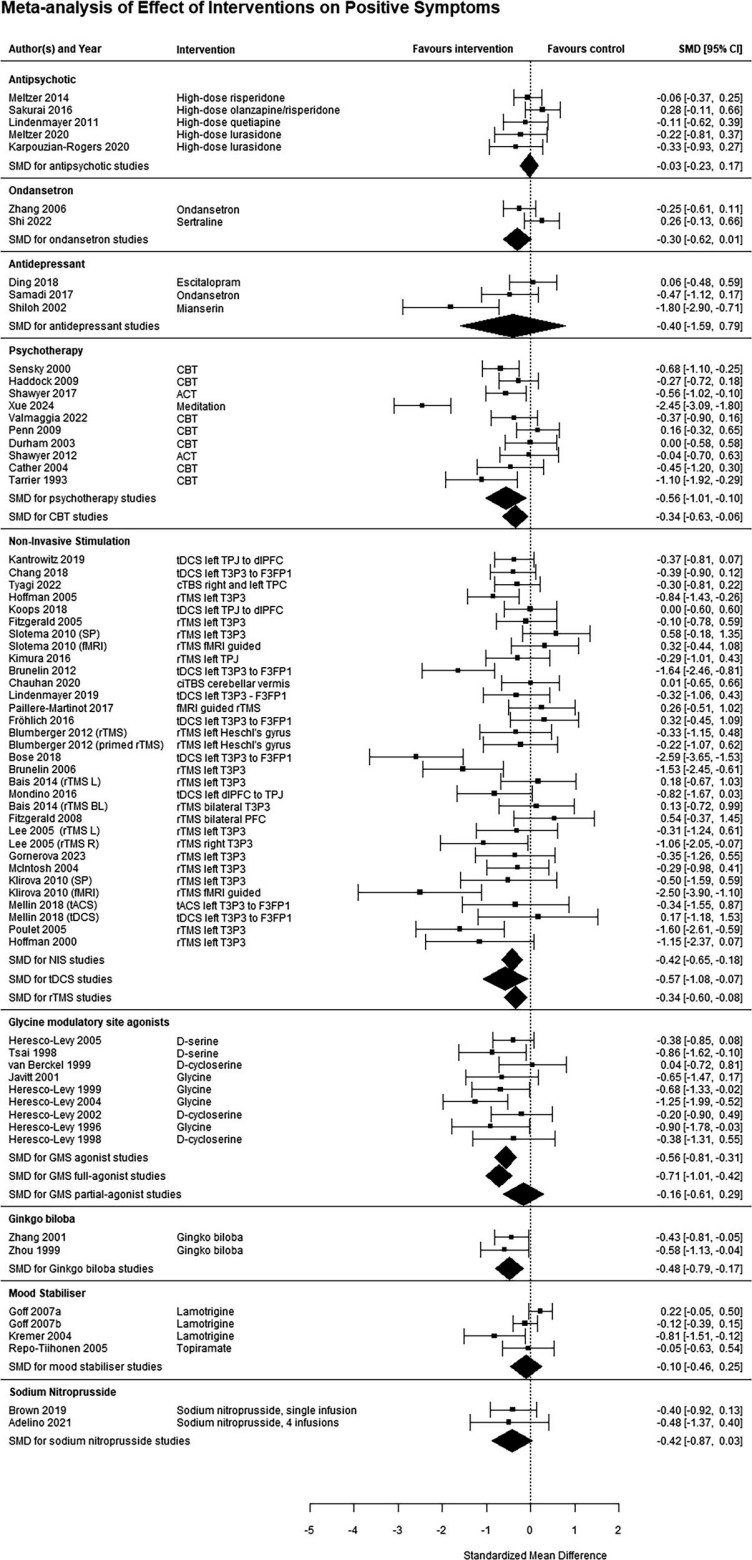
Meta-analysis for effect of interventions on positive symptoms. 5-HT 5-hydroxytryptamine, ACT acceptance and commitment therapy, BL bilateral, CBT cognitive behavioural therapy, CI confidence interval, ciTBS cerebellar intermittent theta burst stimulation, cTBS continunous theta burst stimulation, dlPFC dorsolateral prefrontal cortex, F3 frontal region 3 (10-20 EEG system), fMRI functional magnetic resonance imaging, FP1 prefrontal region 1, HAL haloperidol, L left, NIS non-invasive stimulation, OLA olanzapine, P3 parietal region 3, R right, RIS risperidone, rTMS repetitive transcranial magnetic stimulation, SMD standardised mean difference, SP standard placement, T3 temporal region 3, tACS transcranial alternating current stimulation, tDCS transcranial direct current stimulation, TPJ temporoparietal junction.

**Fig. 3:**
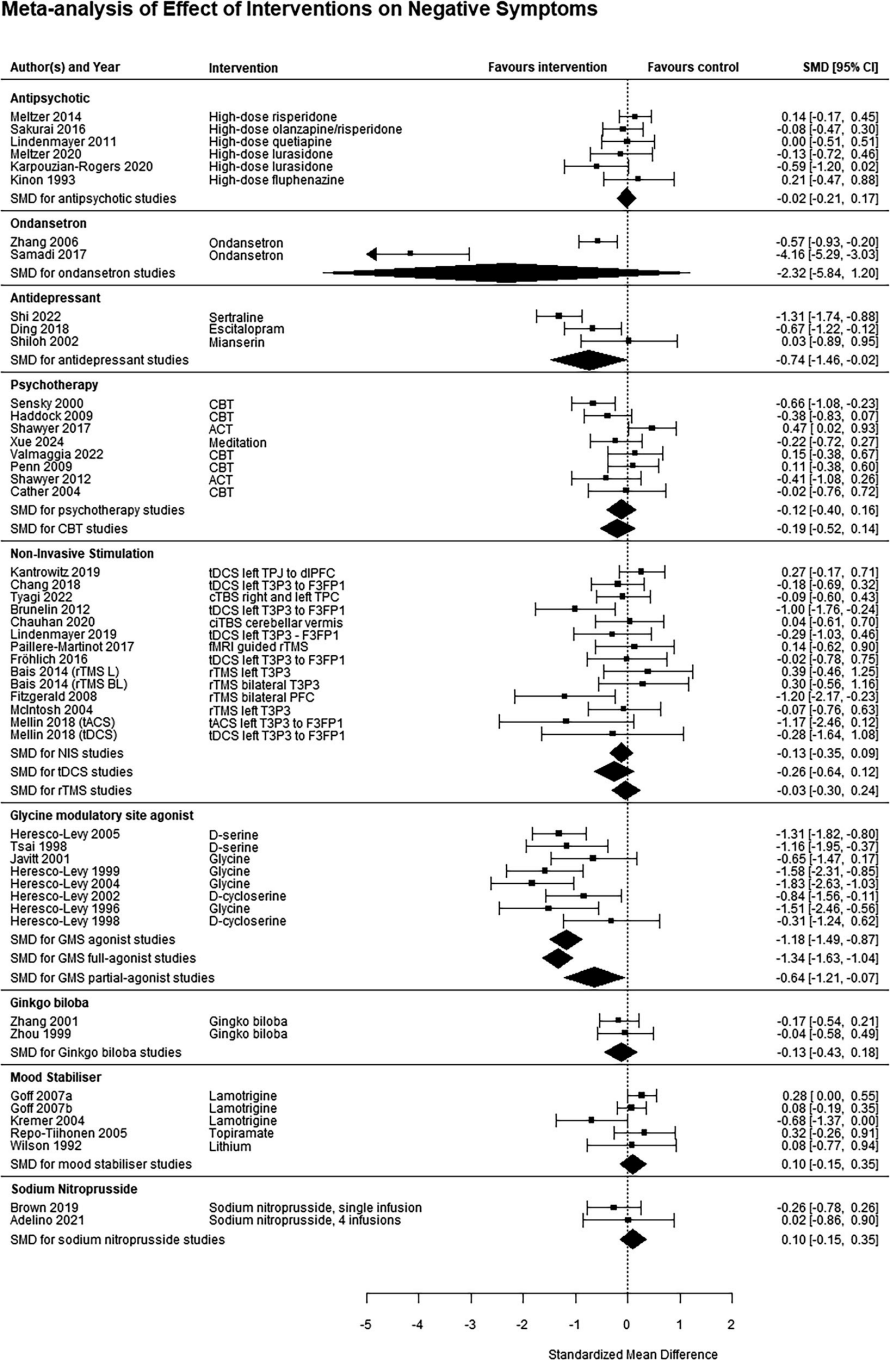
Meta-analysis for effect of interventions on negative symptoms. 5-HT 5-hydroxytryptamine, ACT acceptance and commitment therapy, BL bilateral, CBT cognitive behavioural therapy, CI confidence interval, dlPFC dorsolateral prefrontal cortex, F3 frontal region 3 (10-20 EEG system), fMRI functional magnetic resonance imaging, FP1 prefrontal region 1, HAL haloperidol, L left, NIS non-invasive stimulation, OLA olanzapine, P3 parietal region 3, R right, RIS risperidone, rTMS repetitive transcranial magnetic stimulation, SMD standardised mean difference, SP standard placement, T3 temporal region 3, tACS transcranial alternating current stimulation, tDCS transcranial direct current stimulation, TPJ temporoparietal junction.

**Fig. 4:**
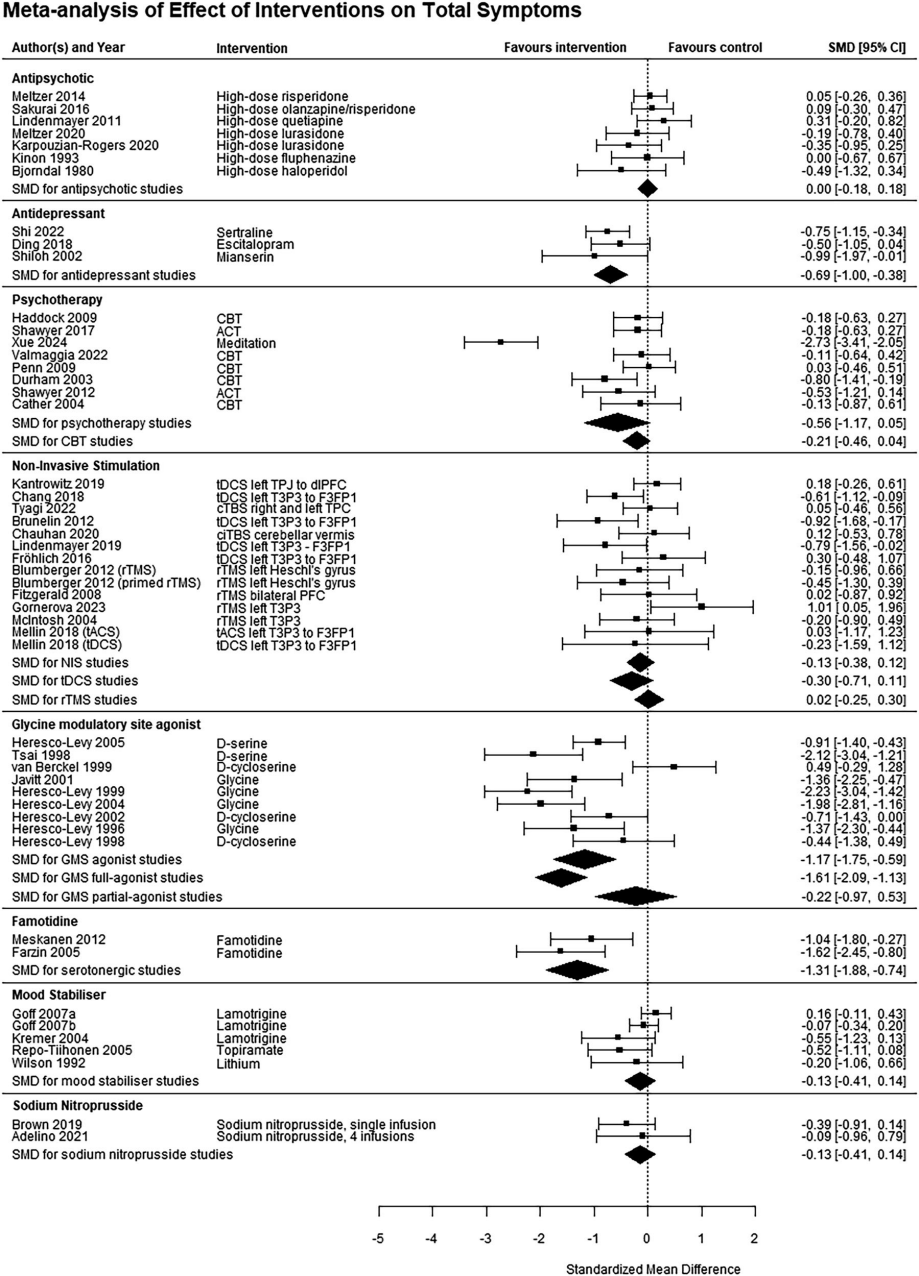
Meta-analysis for effect of interventions on total symptoms. 5-HT 5-hydroxytryptamine, ACT acceptance and commitment therapy, BL bilateral, CBT cognitive behavioural therapy, CI confidence interval, dlPFC dorsolateral prefrontal cortex, F3 frontal region 3 (10-20 EEG system), FP1 prefrontal region 1, HAL haloperidol, L left, NIS non-invasive stimulation, OLA olanzapine, P3 parietal region 3, R right, RIS risperidone, rTMS repetitive transcranial magnetic stimulation, SMD standardised mean difference, SP standard placement, T3 temporal region 3, tACS transcranial alternating current stimulation, tDCS transcranial direct current stimulation, TPJ temporoparietal junction.

Seven studies, comprising 467 subjects [17, 30, 31, 41–44], investigated high-dose antipsychotics compared to standard dose, this involved a parallel group design investigating high vs standard doses of a specific agent. High-dose antipsychotics were defined as doses exceeding doses listed in the British National Formulary. There was no significant benefit for positive (g = −0.03 [−0.23, 0.17], I^2^ = 3.4%, GRADE rating Low), negative (g = −0.02 [−0.21, 0.17], I^2^ = 0%, GRADE rating Low) or total (g = 0.02 [−0.18, 0.18], I^2^ = 0%, GRADE rating Very Low) symptoms; Figs. 2–4).

Nine studies, comprising 187 subjects [22, 25–27, 45–49], investigated drugs targeting the glycine modulatory site (GMS) of the NMDA receptor compared to placebo, including four glycine studies, two d-serine studies and three d-cycloserine studies. A significant benefit, with large effect sizes, was seen for positive (g = −0.56 [−0.81, −0.31], I^2^ = 8.5%, GRADE rating Low), negative (g = −1.18 [−1.49, −0.87], I^2^ = 26.3%, GRADE rating Low) and total (g = −1.17 [−1.75, −0.59], I^2^ = 79.9%, GRADE rating Very Low) symptoms. Subgroup analysis on studies of full agonists of the GMS (glycine and d-serine) remained significant for all three symptom domains, however partial GMS agonists (d-cycloserine) were not effective for any symptom domain (Figs. 2–4*)*. Funnel plots did not show evidence of publication bias (Supplemental eFig. 1). Six of these studies used a crossover design [25–27, 45–47], which have the potential for carry-over effects [50]. However, all included a 2-week washout, 84 times the half-life of glycine [51] and 20 times the half-life of d-serine [52]. Only three of them reported symptom change in the first treatment period [26, 27, 46], and only for negative symptoms. Sensitivity analysis of just the first treatment period of these three studies still found a significant effect on negative symptoms (g = −1.31 [−2.25, −0.37], I^2^ = 83.5%), and the magnitude of the effect size when the three studies without these data were excluded was comparable to the main analysis, albeit with higher heterogeneity (g = −1.14 [−1.71, −0.56], I^2^ = 69.3%).

Five studies, comprising 500 subjects [53–56], investigated mood stabilisers compared to placebo. No significant benefit was found for positive (g = −0.10 [−0.46, 0.25], I^2^ = 68.4%, GRADE rating Low), negative (g = 0.10 [−0.15, 0.35], I^2^ = 36.1%, GRADE rating Low) or total (g = −0.13 [−0.41, 0.14], I^2^ = 47.9%, GRADE rating Low) symptoms (Figs. 2–4).

Five studies, investigated drugs targeting the serotonergic system compared to placebo. These have been split into antidepressants (three studies investigating mianserin [32], sertraline [34] and escitalopram [57], totalling 187 patients) and the 5-HT3 antagonist ondansetron (2 studies [58, 59], 159 patients). Ondansetron did not improve positive (g = −0.30 [−0.62, 0.01], I^2^ = 0%, GRADE rating Very Low) or negative (g = −2.32 [−5.84, 1.20], I^2^ = 97.2%, GRADE rating Very Low) symptoms. Antidepressants significantly improved total (g = −0.69 [−1.00, −0.38], I^2^ = 0%, GRADE rating Low) and negative symptoms (g = −0.74 [−1.46, −0.02], I^2^ = 76.7%, GRADE rating Very Low), with relatively large effect sizes, but not positive symptoms (g = −0.40 [−1.59, 0.79], I^2^ = 91.5%, GRADE rating Very Low, Figs. 2–4).

Two studies each investigated *Ginkgo biloba* [60, 61], sodium nitroprusside [62, 63] and famotidine [64, 65], comprising 163, 80 and 60 patients respectively. *Ginkgo biloba* studies found a significant effect on positive (g = −0.48 [−0.79, −0.17], I^2^ = 0%, GRADE rating Very Low) but not negative (g = −0.13 [−0.43, 0.18], I^2^ = 0%, GRADE rating Low) symptoms. The two famotidine studies showed a significant effect on total symptoms (g = −1.31 [−1.88, −0.74], I^2^ = 3.5%, GRADE rating Low), the only symptom domain reported in both studies (Figs. 2–4). The two sodium nitroprusside studies showed no effect on positive (g = −0.42 [−0.87, 0.03], I^2^ = 0%, GRADE rating Very Low), negative (g = −0.10 [−0.15, 0.35], I^2^ = 0%, GRADE rating Very Low) or total (g = −0.13 [−0.41, 0.14], I^2^ = 0%, GRADE rating Very Low) symptoms.

Ten studies, not fitting into other intervention classes, are reviewed narratively in the Supplemental Methods. Allopurinol [23] was found to be effective for positive, negative and total symptoms in the larger of two crossover studies, although around 1/3 of the subjects withdrew from the trial, many due to inefficacy and no significant findings were reported in the second [24]. Oestradiol showed efficacy for positive and general symptoms in a large and well-designed RCT [66]. Alpha-lipoic acid [67] was found to be effective for negative symptoms. Ketoconazole [68], cholecystekinin octapeptide [69], and the neuropeptide secretin [70] showed no impact on symptom scores, while yokukansan [71] was found to reduce the excitement/hostility items of the PANSS only. ECT showed a small benefit over placebo for total symptoms, and significantly reduced readmission rates [72]. Bumetanide showed efficacy for audioverbal hallucinations in a small RCT [73].

### Non-invasive stimulation

26 studies, comprising 893 subjects [28, 29, 74–97], including three crossover trials, investigated non-invasive stimulation compared to sham stimulation. Nine of these [29, 76, 77, 79, 80, 84, 86, 89, 96] investigated tDCS or transcranial alternating current stimulation (tACS), and the rest investigated rTMS or theta burst stimulation [95, 97]. With the exception of five studies, these investigated rTMS stimulating the left or bilateral temporo-parietal junction and tDCS or tACS applying stimulation between the left temporo-parietal junction (TPJ) and frontal cortex. Three studies [85, 90, 92] used fMRI-guided rTMS, either in addition to or instead of standard temporo-parietal placement; these still delivered stimulation to the temporo-parietal junction, but precise placement was determined via fMRI activations during hallucinatory perception.

For non-invasive stimulation studies as a whole, a significant benefit was found for positive symptoms, with a moderate effect size (g = −0.42 [−0.65, −0.18], I^2^ = 64.9%, GRADE rating Low), but not total (g = −0.13 [−0.38, 0.12], I^2^ = 37.7%, GRADE rating Very Low) or negative (g = −0.13 [−0.35, 0.09], I^2^ = 23.9%, GRADE rating Very Low; Figs. 2–4) symptoms. For rTMS studies, a significant benefit was found for positive (g = −0.34 [−0.60, −0.08] I^2^ = 55.2%), but not negative (g = −0.03 [−0.30, 0.24], I^2^ = 0%) or total (g = −0.02 [−0.25, 0.30], I^2^ = 0%) symptoms. For tDCS/tACS studies, a significant benefit was found for positive (g = −0.57 [−1.08, −0.07], I^2^ = 78.0%), but not negative (g = −0.26 [−0.64, 0.12], I^2^ = 46.9%) or total (g = −0.30 [−0.71, 0.11], I^2^ = 52.8%) symptoms.

There was a wide range in the duration of treatment in NIS studies, from 2 consecutive weekdays [28] to 20 [29, 75]. However, meta-regressions did not show evidence that duration of treatment moderated symptom change in the positive (β = 0.03, p = 0.31), negative (β = −0.03, p = 0.32) or total (β = −0.01, p = 0.51) domains.

Funnel plots did show evidence of asymmetry (Supplemental eFig. 2), and Egger’s test for asymmetry was significant (z = −2.29, p = 0.022). When potentially missing studies were imputed via trim and fill analyses the effect size for positive symptoms became non-significant (g = −0.18 [−0.47, 0.12]). The same was true when limited to rTMS studies (g = −0.14, [−0.46, 0.18]).

### Psychotherapy

Ten studies, comprising 565 subjects [18–21, 35, 36, 38–40, 98], investigated psychotherapy using various comparators such as befriending or supportive counselling. Multiple trials investigated the effect of two different psychotherapy models, namely cognitive behavioural therapy [18–21, 36, 39, 40] and acceptance and commitment therapy [35, 37]. One study investigated structured intensive meditation [38]. Psychotherapy was found to be effective for positive (g = −0.56 [−1.01, −0.10], I^2^ = 85.2%, GRADE rating Low), but not negative (g = −0.12 [−0.40, 0.16], I^2^ = 57.0%, GRADE rating Low) or total (g = −0.56 [−1.17, 0.05], I^2^ = 89.6%, GRADE rating Low) symptoms (Figs. 2–4*)*. Funnel plots did not show evidence of publication bias (Supplemental eFig. 3), and Egger’s test was not significant (z = −0.94, p = 0.35).

In order to try and reduce heterogeneity, an analysis only considering CBT studies was performed. This found that a benefit for positive symptoms was maintained (g = −0.34 [−0.63, −0.06], I^2^ = 45.9%), with reduced heterogeneity, but there remained no significant benefit for negative or total symptoms (Figs. 2–4).

### Acceptability

All interventions, with the exception of high-dose antipsychotics, were generally well-tolerated. A review of intervention acceptibility is presented in the Supplementary Materials.

### Meta-regressions

Meta-regressions were performed for NIS and psychotherapy studies only, where there were more than 10 studies in the intervention class reporting the relevant information, as per Cochrane guidelines. There was no significant effect of baseline symptom severity, proportion of patients taking clozapine, antipsychotic dose or the proportion of patients with a diagnosis of schizoaffective disorder on effect size in any symptom domain (see Supplementary Materials).

### Wald-type tests

In most intervention classes, data were insufficient to explore reasons for heterogeneity, however Wald-type tests were performed to investigate the impact of bias and TRS definition on results. Risk of bias did not affect the effect size in any symptom domain. Effect size was significantly different between NIS studies using a lenient and strict TRS definition in the positive symptom domain (z = 2.04, p = 0.04), but not for other symptom domains, or any symptom domains in psychotherapy or antipsychotic studies. These results, the methods for calculating them, and sensitivity analyses exploring them further, are reported in more detail in the Supplement.

### Effect of allegiance bias on results

Significant conflicts of interest were identified for 12 studies included in the meta-analysis. Sensitivity analyses excluding these studies did not change results (see Supplement), except in GMS studies, where 7 studies were excluded, and the effect became nonsignificant for positive (g = −0.41 [−1.3, 0.47]) and total symptom (g = −0.80 [−3.37, 1.76]) domains. The effect on negative symptoms remained significant (g = −1.16 [−1.54, −0.60]). However, only two studies were included in this sensitivity analysis.

### Effect of correlation coefficient on results

We imputed change scores using a correlation coefficient of 0.74, an average from 4 studies (see Supplemental Methods for calculation). A sensitivity analysis using a conservative correlation coefficient of 0.5 [99] did not affect the significance of our results, with the exception of the partial GMS agonist subset of studies in the negative symptom domain, which became non-significant (g = −0.47, [−1.3, 0.10]).

### GRADE assessments

GRADE assessments were performed for each outcome reported. The Summary of Findings table is presented in Table 2. The detailed rationale for each outcome rating is available in the Supplemental Materials.

**Table 2:** Summary of Findings table describing the GRADE assessment ratings for each outcome.

Outcome	N; patients (studies)	Effect [CI] (significant in bold)	RoB (number of studies)			I^2^	Indirectness (%meeting TRS criteria)	Imprecision (number of thresholds crossed)	Publication bias risk	GRADE Rating
			L	M	H					
Antipsychotic, **positive**	410 (5)	−0.03 [−0.23, 0.17]	1	0	4	3.4%	60%	2, inc. null	Low	* *
Antipsychotic, **negative**	444 (6)	−0.02 [−0.21, 0.17]	1	0	5	0%	50%	2, inc. null	Low	* *
Antipsychotic, **total**	467 (7)	0.00 [−0.18, 0.18]	1	0	6	0%	43%	1, inc. null	Low	*
Ondansetron, **positive**	159 (2)	−0.30 [−0.62, 0.01]	1	0	1	0%	0%	3, inc. null	NA	*
Ondansetron, **negative**	159 (2)	−2.32 [−5.84, 1.20]	1	0	1	97.15%	50%	7, inc. null	NA	*
Antidepressant, **positive**	187 (3)	−0.40 [−1.59, 0.79]	2	0	1	91.5%	100%	6, inc. null	NA	*
Antidepressant, **negative**	187 (3)	−**0.74 [**−**1.46**, −**0.02]**	2	0	1	76.7%	100%	3	NA	*
Antidepressant, **total**	187 (3)	−**0.55 [**−**0.89**, −**0.22]**	2	0	1	0%	100%	2	NA	* *
Psychotherapy, **positive**	565 (10)	−**0.56 [**−**1.01**, −**0.10]**	3	4	3	85.2%	60%	3	Low	**
Psychotherapy, **negative**	493 (8)	−0.12 [−0.40, 0.16]	3	4	1	57.0%	50%	2, inc. null	Low	* *
Psychotherapy, **total**	493 (8)	−0.56 [−1.17, 0.05]	2	4	2	89.6%	62.5%	4, inc. null	Low	* *
NIS, **positive**	893 (26)	−**0.42 [**−**0.65**, −**0.18]**	15	5	6	64.9%	73%	2	High	* *
NIS, **negative**	443 (12)	−0.13 [−0.35, 0.09]	6	3	3	23.9%	67%	2, inc. null	High	*
NIS, **total**	443 (12)	−0.13 [−0.38, 0.12]	8	1	3	37.7%	67%	2, inc. null	High	*
GMS agonist, **positive**	187 (9)	−**0.56 [**−**0.81**, −**0.31]**	5	1	3	8.5%	88.9%	2	Some	* *
GMS agonist, **negative**	161 (8)	−**1.18 [**−**1.49**, −**0.87]**	4	1	3	26.3%	100%	0	Some	* *
GMS agonist, **total**	187 (9)	−**1.17 [**−**1.75**, −**0.59]**	5	1	3	79.9%	88.9%	1	Some	*
*Gingko biloba*, **positive**	163 (2)	−**0.48 [**−**0.79**, −**0.17]**	1	0	1	0%	100%	2	NA	*
*Gingko biloba*, **negative**	163 (2)	−0.13 [−0.43, 0.18]	1	0	1	0%	100%	2, inc. null	NA	* *
Famotidine, **total**	60 (2)	−**1.31 [**−**1.88**, −**0.74]**	1	1	0	3.5%	100%	1	NA	*
Mood stabiliser, **positive**	479 (4)	−0.10 [−0.46, 0.25]	3	1	0	68.4%	25%	3, inc. null	Low	* *
Mood stabiliser, **negative**	500 (5)	0.10 [−0.15, 0.35]	4	1	0	36.1%	20%	2, inc. null	Low	* *
Mood stabiliser, **total**	500 (5)	−0.13 [−0.41, 0.14]	4	1	0	47.9%	20%	2, inc. null	Low	* *
Sodium nitroprusside, **positive**	80 (2)	−0.42 [−0.87, 0.03]	0	2	0	0%	50%	4, inc. null	NA	*
Sodium nitroprusside, **negative**	80 (2)	−0.10 [−0.15, 0.35]	0	2	0	0%	50%	2, inc. null	NA	*
Sodium nitroprusside, **total**	80 (2)	−0.13 [−0.41, 0.14]	0	2	0	0%	50%	2, inc. null	NA	*

*CI* confidence intervals, *RoB* risk of bias, *L* low (risk of bias), *M* medium (risk of bias), *H* high (risk of bias).

For the ratings, * very low, **low, *** moderate, **** high, ***** very high.

## Discussion

In this systematic review and meta-analysis, we investigated the efficacy of nine classes of non-clozapine interventions to treat TRS. The main findings were the significant efficacy of NIS, psychotherapy, serotonergic, and especially GMS agonist interventions for various symptom domains; it should be noted that all RCTs described here delivered interventions in addition to standard therapy, and our findings should be interpreted as augmentation strategies, rather than alternatives to antipsychotic treatment.

High-dose antipsychotics are a common treatment strategy in TRS, with a third of individuals receiving antipsychotic therapy above the maximum licensed dose before starting clozapine [100]. Our findings show no benefit of high doses over standard doses in managing any symptom domain, but due to the high risk of bias, and the fact that the majority of studies investigated different agents, the evidence is of only low certainty. Missing data represents a significant problem for these studies; high-dose antipsychotics carry heavy side effect burdens [101] (see Supplement), which in one study led to a withdrawal rate in the high-dose group 10x that of the standard dose group [42]. In such cases, a last-observation-carried-forward approach may help account for this, but this was reported in only one study [30]. The result of this is that our findings are likely biased in favour of high-dose antipsychotics, as patients who experienced symptomatic worsening were less likely to be included in the analysis. This is less problematic for other interventions which do not carry such high side effect burdens.

Nonetheless, our findings are in line with Cochrane evidence that increasing the dose of antipsychotics does not benefit people who have not shown response to their initial treatment [102], and indicate that high-dose treatment may not be an effective general strategy for TRS, although we cannot exclude the possibility that it might benefit selected patients. Our finding are consistent with imaging studies that suggest presynaptic dopamine is not increased in psychosis unresponsive to D2 antagonists, suggesting a different therapeutic mechanism is required to treat TRS [103]. An alternative explanation is that higher doses of antipsychotics may show diminishing returns in terms of clinical efficacy. The therapeutic window for D2 antagonists sits at between 60–80% D2 receptor occupancy, with higher occupancies conferring little additional benefit on average. However, with some antipsychotics, meta-analytic evidence suggests the dose-response curve does not reach a plateau at standard therapeutic doses [104]. We did not include any studies investigating sertindole, ziprasidone or iloperidone, three antipsychotics where there the possibility of higher-than-standard doses being more efficacious has not been excluded [104].

GMS modulators showed significant benefit in all symptom domains, with large effect sizes and low heterogeneity for positive symptoms. The effect on positive symptoms in particular was driven by glycine and d-serine, which are full endogenous agonists at the GMS; d-cycloserine, a partial agonist, did not show any benefit for positive symptoms. The three compounds share similar mechanisms. Glycine and d-serine are full endogenous agonists at the glycine modulatory site of the NMDA receptor, enhancing glutamatergic signalling [105]. Meanwhile, d-cycloserine is a exogenous partial agonist [106], and while it also enhances NMDA function with around 40–50% the efficacy of glycine [107], it will act in competition with endogenous glycine and d-serine, with higher doses leading to net downregulation of NMDA-dependent signalling [107]. In line with this, two open-label studies of high-dose d-cycloserine (250–500 mg daily) in schizophrenia found significant deterioration of psychotic symptoms [108, 109]. This may explain the inefficacy observed in our analysis, and the fact that the effect size of the three d-cycloserine studies inversely relates to administered dose. However, all of the studies were small, and with all except two originating from a single research group, which led to downgrading the certainty of the evidence to Low-to-Very-Low. We should also note that one of the authors in 7 of the studies [25–27, 45–47, 110] acquired intellectual property rights for the use of d-serine in treating psychosis in 2000. Excluding all studies on which he was an author left only two studies, and only the significant effect on negative symptoms survived sensitivity analysis. Prior research suggests that adding glutamatergic agents to clozapine shows only a minor benefit in terms of positive symptoms [111]. There is evidence that clozapine has glutamatergic activity [112], which may diminish the benefit from additional glutamatergic modulation. One other explanation for the difference between our findings and the lack of effect with clozapine augmentation with glycine or d-serine is that while TRS is associated with a treatable glutamatergic pathophysiology, the same is not true for clozapine-resistant schizophrenia [113]. Also in contrast to our finding, augmentation with glutamatergic medications in people with schizophrenia not selected for TRS does not show a convincing benefit [114], suggesting that their efficacy may be specific to TRS, in keeping with findings that there are distinct glutamatergic alterations in TRS compared to D2 antagonist-responsive schizophrenia [115–117], which is supported by meta-analytic work showing greater glutamatergic neurometabolite levels in the cingulate cortex [118]. Funnel plots did not suggest publication bias, however this is based on a limited sample. While the evidence base is limited, our findings do warrant further investigation in larger RCTs.

Famotidine and *Ginkgo biloba* extract showed efficacy for total and positive symptoms respectively, however both of these classes were limited to two studies, and the GRADE rating for both outcomes was Very Low due to the number of studies and issues surrounding bias (see Supplement). As a result, no firm recommendations can be made, except for further pilot studies to inform the design of larger RCTs in the future.

NIS showed a small overall benefit in positive symptoms. Both tDCS and rTMS were found to be effective for auditory hallucinations, but with high study heterogeneity, likely partly due to a differential effect on people meeting the TRRIP criteria for TRS, and significant concerns regarding publication bias, which led us to rate the certainty of this evidence as low. Residual heterogeneity after accounting for the TRS definition used may result from between-study differences in stimulation parameters, length and frequency of stimulations, and rating scales. Many studies used rating scales specific to auditory verbal hallucinations, e.g., the Auditory Hallucination Rating Scale (AHRS), and not the PANSS; as such, the effect on positive symptoms should be interpreted primarily as an effect on hallucinations. No significant effect was found for total or negative symptoms. However, with the exception of one study [82], the vast majority of NIS studies focused on explicitly treating auditory verbal hallucinations, and as such stimulated the TPJ in the case of rTMS and between the frontal cortex and TPJ in tDCS [119–121]. Previous tDCS studies targeting negative symptoms tended to place both the cathode and anode over frontal regions [119, 120], or in the case of rTMS, stimulation is applied to the dlPFC [121]. As such, from this analysis we cannot conclude that rTMS and tDCS are ineffective for negative symptoms; rather only that negative symptoms are unlikely to be well-treated by non-invasive stimulation targeting hallucinations. The duration of treatment in NIS studies was generally very short, with some studies only giving treatment for a few days, and the majority administering 10 sessions over consecutive weekdays; as such, these findings should be interpreted as the acute effects of short-term treatment. However, we did not find evidence for an effect of treatment duration on symptom change in meta-regression.

NIS studies, however, showed significant asymmetry in funnel plots, and the improvement in positive symptoms became non-significant following trim-and-fill analysis. In addition, although the significant benefit was maintained when limiting analysis to studies using TRS criteria meeting the TRRIP consensus, there was significantly lower effect size in studies using a more conservative TRS definition, raising concerns that our results may not reflect a true clinical benefit, especially in patients meeting the TRRIP criteria for TRS. As such, we downgraded the GRADE rating for this outcome to Low.

Previous meta-analytic work has found that psychotherapy, in the form of CBT, was effective for positive symptoms of TRS [122, 123]. Our results corroborate these findings with a consistent effect size, however the finding of an effect of psychotherapy on total symptoms in prior work was not replicated [122]. This may be due to the different inclusion criteria; our analysis excluded studies using TAU as a control condition and trials, which had been included in prior analyses, without any requirements for non-response to antipsychotics. Excluding TAU is likely to have moderated the effect size (see Methods), potentially explaining the lack of effect we observed on total symptoms. One interpretation of these findings is that CBT does have a specific effect on positive symptoms of schizophrenia not seen in active controls, but an effect on general symptoms may relate to the social interaction of psychotherapy which may also be achieved through befriending or supportive counselling, which themselves are still superior to no intervention. Psychotherapy studies showed high heterogeneity and carried high RoB due to the impossibility of blinding, leading us to downgrade the GRADE rating for this outcome to Low. When limited to CBT, thus excluding one outlier study which showed a large effect of meditation on positive symptoms [38], heterogeneity greatly diminshed, and a modest benefit for positive symptoms was maintained, suggesting the type of therapy may explain the observed heterogeneity.

Mood stabilisers are a common augmentation strategy in TRS, and limited meta-analytic evidence does support their use in managing positive symptoms when added to clozapine [53, 124]. However, we did not find such a benefit. Indeed, there is evidence that mood stabiliser augmentation is more effective when combined with clozapine compared to other antipsychotics [125], which may explain our findings. However, only one study in this group met the TRRIP criteria, which limits the conclusions that can be drawn to the treatment-resistant population.

Ondansetron, a selective 5-HT_3_ antagonist, was not effective in the positive or negative domains, however we only found two studies in TRS, one of which was of dubious quality and showed an extremely large effect on negative symptoms [59], and our GRADE assessment rating for this group was Very Low. Previous meta-analyses investigating ondansetron in a general schizophrenia cohort found a significant effect on total symptoms [33, 126], which we were unable to assess in our analysis. Further large, well-designed RCTs are needed before any recommendations can be made for their clinical use.

Antidepressants were found to improve total and negative symptoms, and although only three studies were included, these studies were generally well-designed and consistent in the total symptom domain. However, heterogeneity was still substantial, and with negative symptoms, results were imprecise, leading to a GRADE assessment rating of Low to Very Low. The significant effect on total scores is likely to result from improvements in mood and anxiety symptoms. An improvement in negative symptoms in TRS adds to previous meta-analytic evidence in treatment-responsive [127], chronic [128] and clozapine-resistant [129] schizophrenia which reported similar findings. However, all antidepressant studies used the PANSS (Table 1), which has received some criticism as a measure of primary versus secondary negative symptoms [130] and in differentiating these from depressive symptoms [131]. It is therefore difficult to claim that improvements in PANSS negative subscale scores were not related to improvement in general psychopathology. It should also be noted that the antidepressants group contained studies on drugs with different mechanisms; mianserin is a 5-HT2a antagonist, while citalopram and sertraline are selective serotonin reuptake inhibitors. While both of these drug classes show efficacy for depressive symptoms [132], it cannot be excluded that they have different interactions with non-affective illness; especially since mianserin shows pharmacodynamic overlap with some antipsychotic medications such as olanzapine and clozapine [133], despite lacking antidopaminergic activity.

Narrative review of lone studies suggest that oestradiol may be a promising intervention in TRS, based on positive findings in one large and well-designed RCT [66]. Epidemiological evidence supports a protective role for oestrogen against psychotic symptoms; [134] it has been noted that positive symptoms fluctuate with the course of the menstrual cycle [135], and in the postpartum period, when oestrogen levels drop dramatically, women are at a significantly higher risk of developing psychosis [136], both in the context of first episode or relapse of existing illness. Further studies of hormonal interventions in TRS are warranted. Electroconvulsive therapy is a common intervention in TRS, however the majority of RCTs have been performed as augmentation to clozapine. We found only one study meeting the inclusion criteria, which showed a mild benefit. Zheng et al. have performed a meta-analysis of ECT as non-clozapine antipsychotic augmentation in TRS [137], finding a significant benefit, however only one study, which we have discussed narratively, included a sham comparator. Further sham-controlled RCTs are warranted to determine the efficacy of ECT when added to non-clozapine antipsychotics.

### Limitations and future directions

The primary limitations of the trials included in this analysis were the relatively small study size across interventions, and publication bias, which could only be rigorously assessed for NIS studies, which did show evidence of bias. As such, while results are not conclusive, they do point towards some promising avenues for future larger, well-designed trials of interventions in TRS, in particular GMS agonists, with the caveat that the paucity of GMS agonist studies outside of a single research group made assessing the effect of allegiance bias on this intervention challenging. Iclepertin, a glycine transporter 1 inhibitor, is currently in phase III trials and has shown promise in treating cognitive symptoms of the illness; studying its effects in treatment resistance would be valuable. A novel antipsychotic, xanomeline/trospium, which targets the muscarinic system, has also shown promise in schizophrenia [138], but has not yet been studied in TRS.

The duration of treatment in NIS studies in particular was very short, although we did not find evidence that treatment duration affected symptom change. Future studies should aim to determine whether longer treatment with NIS leads to greater improvements in symptoms, and whether maintenance therapy is needed to sustain clinical benefits.

Although this analysis excluded RCTs explicitly investigating clozapine augmentation, most of the studies employed cohorts that included some patients on clozapine, and likely individuals who may have received clozapine in the past. Although the relative uniqueness of clozapine’s pharmacological profile is not entirely clear [139, 140], it has been found, for example, that augmentation of clozapine with an mood stabilisers leads to greater additive effects compared to augmentation of non-clozapine antipsychotics [125]. Our results cannot be extended to clozapine-resistant schizophrenia, which is likely to have a different neurobiology to both treatment-responsive schizophrenia and TRS. However, it is possible that several patients in the sample did meet the criteria for this, although it was rarely reported in clinical assessments, likely adding further heterogeneity to our results. Previous meta-analytic work has suggested efficacy for augmentation of clozapine with mood stabilisers, electroconvulsive therapy and antidepressants [129], however it remains unclear what options, other than electroconvulsive therapy, are appropriate in such patients who cannot take clozapine.

The papers in this review made no distinction between primary and secondary negative symptoms; as such, interventions where both positive and negative symptoms responded to the intervention should be interpreted cautiously.

There is wide inter-individual variability in response to dopamine receptor antagonists [141] and the same may well be the case of augmentation interventions. Identifying which patient will benefit from which treatment strategy has the potential to markedly increase efficacy. For example, studies investigating whether the effect of glutamatergic interventions is greater in patients showing glutamatergic dysfunction on magnetic resonance spectroscopy, could help personalise interventions. Related to this is the question as to whether the baseline antipsychotic prescribed affects the efficacy of augmentation. This highlights the need for head-to-head comparisons of non-clozapine interventions with clozapine using glutamatergic modulators, NIS, and psychotherapy.

Finally, our classification of interventions may have introduced some noise into the findings. For antidepressants, high-dose antipsychotics and mood stabilisers, drugs with different mechanisms were included in the same subgroups. For example, there is meta-analytic evidence of differential efficacy of antipsychotics in TRS, with olanzapine in particular emerging favourably versus other non-clozapine antipsychotics [142]. As such, negative results from these three classes does not exclude the possibility that specific agents may show efficacy.

### Conclusions

This meta-analysis suggests that high dose antipsychotics may not be effective in the management of TRS. Our findings also suggest that NIS, psychotherapy, and GMS agonists may show efficacy for treatment resistant positive symptoms when clozapine treatment is not indicated, although publication bias and potential alliegance bias limits conclusions. In addition, antidepressants and GMS agonists may show benefit for negative symptoms.

## Supplementary information

Supplement

eFigure 4
