# Supplementary material for: Non-Clozapine interventions in treatment-resistant schizophrenia: a systematic review and meta-analysis
Source: Mol Psychiatry. 2025 Oct 3;31(1):526–44. doi: 10.1038/s41380-025-03255-y (PMC12700803; doi:10.1038/s41380-025-03255-y)
Supplement: Supplementary file 1 — Supplement [file 41380_2025_3255_MOESM1_ESM.docx]

Supplemental Content

1. **Supplemental methods**
2. **eFigure 1** – Funnel plots for glutamatergic studies
3. **eFigure 2** – Funnel plots and trim-and-fill plot for NIS studies.
4. **eFigure 3 –** Funnel plot for psychotherapy studies.
5. **Narrative review of studies not included in meta-analysis**
6. **Review of intervention acceptibility**
7. **eFigure 4** – Breakdown of RoB scores for each study in each RoB domain.
8. **Results of meta-regressions**
9. **Results of Wald-type tests** **of the impact of RoB and TRS definition on effect size**
10. **Results of sensitivity analyses excluding studies with significant conflicts of interest**
11. **GRADE assessment rationale for each outcome**
12. **References**
13. **Supplemental methods**

*Exclusion of studies investigating standard dose antipsychotics in TRS*

Studies using standard-dose antipsychotics as the intervention group were excluded. This was done for two reasons: firstly, several meta-analyses in the past have looked at this question^1-4^, and secondly, the vast majority of standard-dose RCTs lacked a placebo comparator due to ethical reasons, and instead compared to other antipsychotics as separate interventions, precluding comparisons to other interventions analysed in this paper. Instead, we decided to include studies investigating high- versus standard-dose antipsychotics, where two separate arms investigating different doses of the same antipsychotic could be compared against each other.

*Calculating effect-size for crossover trials*

Effect size for crossover trials was calculated by treating the changes in rating scale scores during intervention and placebo treatment as two parallel groups. While this method is susceptible to unit-of-analysis error^5^, it is a conservative method of handling data from crossover studies.

*Studies investigating two separate interventions or intervention parameters*

Several studies investigated different interventions (e.g. transcranial alternating current stimulation vs transcranial direct current stimulation), different parameters of the same intervention (e.g. different doses of the same drug, or different stimulation locations in rTMS studies). In these cases, the two interventions were analysed separately compared to the same placebo group, with n of the placebo group halved to avoid over-weighting.

*Risk of bias assessment*

The Cochrane RoB tool (v2) was used to assess for risk of bias.

As many studies did not report on whether analysis was carried out according to a pre-specified analysis plan, we rated studies as having low RoB in item 5 if they reported results for all outcome measures described in the Methods section, and if those outcomes seemed likely to have been pre-specified. While many studies did not use an intention-to-treat analysis method, these studies were still rated as having a low risk of bias for item 2 if no participants withdrew from the study, as there would be no scope for post-randomisation exclusion related to allocation.

The full risk of bias assessment for each study can be viewed in Supplementary Table 1.

*Imputing standard deviations in change scores from baseline and follow-up*

Most studies did not report improvement as change in rating scale scores with SD, rather as baseline and follow-up rating scale scores pre- and post-intervention. To extract mean change scores for use in our analysis, we subtracted the baseline score from the follow-up score. We imputed SD using the following formula^5^:

$$\sqrt{{((followup SD)}^{2}+{(baseline SD)}^{2})-(2\times0.73\times\left( followup SD \right)\times\left( baseline SD \right))}$$

0.73 was used as the correlation coefficient ($r$) between baseline and follow-up for imputing SD. This was calculated from 4 studies^6-9^ which reported baseline, follow-up and change score SD, using the following formula:

$$r=\frac{({baseline SD}^{2}+{followup SD}^{2}-{change SD}^{2})}{(2\times baseline SD \times followup SD)}$$

1. **
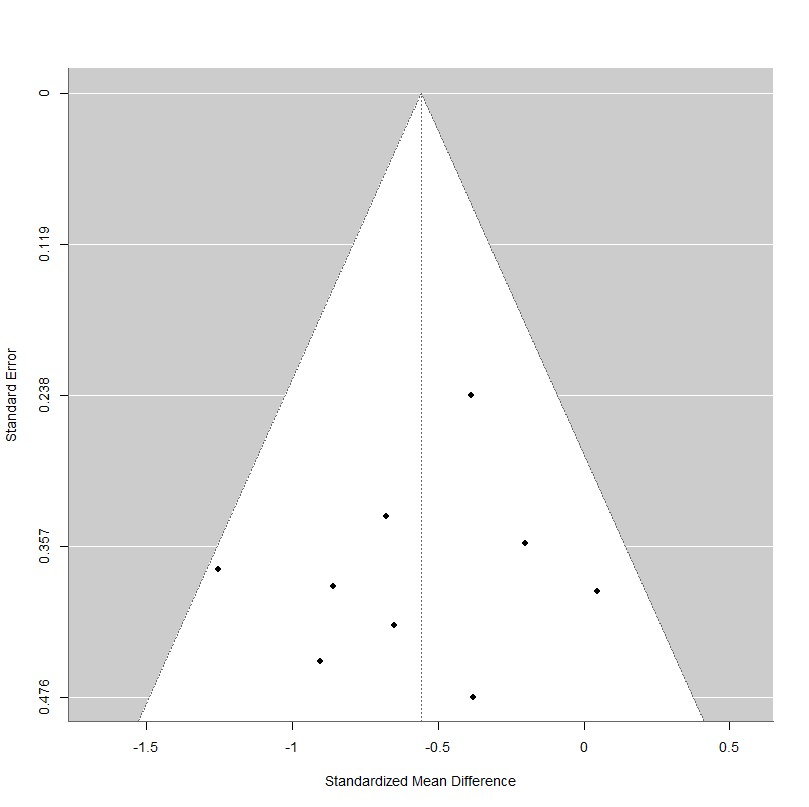
eFigure 1** – Funnel plot for positive symptoms in studies investigating glutamatergic drugs.
2. **eFigure 2** - Funnel plot and funnel plot with trim-and-fill analysis for positive symptoms in studies investigating non-invasive stimulation. Egger’s test was significant (z = -2.29, p = 0.022).

**
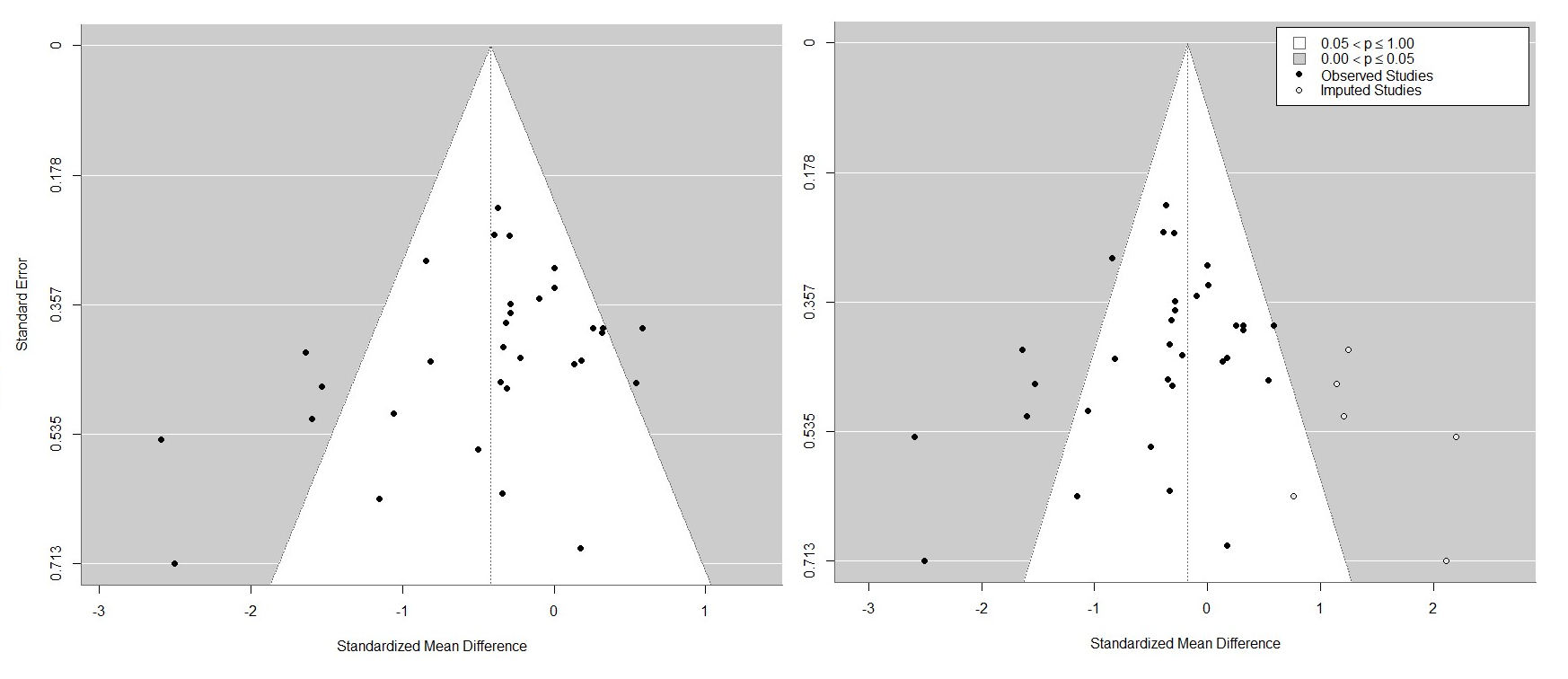
**

1.
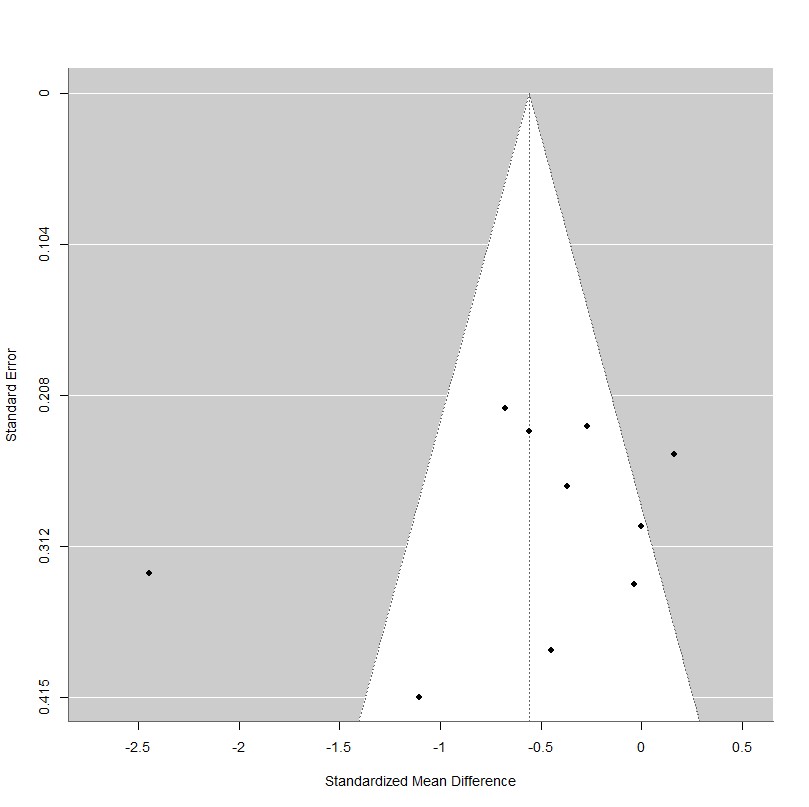
**eFigure 3** – Funnel plot for positive symptoms in studies investigating psychotherapy. Egger’s test was not significant (z = -0.94, p = 0.35).
2. **Narrative review of studies not included in meta-analysis**

Brunstein et al. (2005)^10^ studied allopurinol therapy (300mg BD, orally) in a cross-over design. 12 of the 35 subjects withdrew over the course of the study, the majority due to lack of efficacy. The drug was well-tolerated, and repeated-measures MANOVA suggested significant improvement in positive (-21% ± 17% change in allopurinol vs +11% ± 42% change in placebo group, p <0.001), negative (-8% ± 13% change in allopurinol vs +7% ± 16% change in placebo group, p <0.001), and total (-15% ± 12% change in allopurinol vs +7% ± 20% change in placebo group, p <0.001) PANSS scores, despite carryover effects into the placebo phase observed in patients who responded to allopurinol.

Goswami et al. (2003)^11^ studied electroconvulsive therapy in 25 patients with TRS, with 10 receiving sham stimulation and 15 receiving ECT, added to oral chlorpromazine up to 1000mg/d. Both groups showed numerically similar declines in BPRS scores (55 to 44 in ECT group, 50.1 to 40.4 in the sham group). The authors report only the ECT group showed a statistically significant difference (p <0.002 vs p = 0.542). No significant difference in change in CGI scores was detected. They also found significantly lower readmission rates in the treatment group (70% vs. 20%). Side effects were not reported.

Kulkarni et al. (2015)^12^ studied transdermal oestradiol in women with schizophrenia or schizoaffective disorder. 59 participants received 100mcg/day, 62 received 200mcg/day of oestradiol, and 62 participants received placebo via transdermal patch. A significant benefit of oestradiol was observed in the positive (PANSS positive change: -1.82 in placebo, -2.82 in oestradiol 100mcg, -4.13 in oestradiol 200mcg) and general (PANSS general change: -2.5 in placebo, -5.63 in oestradiol 200mcg) symptom domains. The only adverse effect was a higher rate of irregular menses in the oestradiol 200mcg group (68% in oestradiol 200mcg vs 38% in placebo).

Marco et al. (2002)^13^ studied ketoconazole, up to 800mg/d, as an antiglucocorticoid agent in patients with schizophrenia. 8 subjects received ketoconazole, while 7 received placebo. No significant differences in any PANSS subscale was observed over the course of the study. The only adverse effect noted was mild dizziness in the ketoconazole group (63% in ketoconazole vs 29% in placebo).

Mishra et al. (2022)^14^ studied alpha-lipoic acid (ALA), an antioxidant that may upregulate NMDA receptor expression as augmentation of regular antipsychotics. 10 subjects received ALA, 9 received placebo. A significant difference in SANS (p = 0.007) but not SAPS (p = 0.780) scores was found in the ALA group versus placebo. ALA was well-tolerated.

Miyaoka et al.(2015)^15^ studied extract of yokukansan (YKS), a traditional Japanese remedy shown to have psychopharmacological activity as a D2/5-HT1a partial agonist and a 5-HT2a and glutamate antagonist, given orally for 4 weeks. Given its exotic pharmacological profile, this study was not included in any one intervention class. 56 of 120 subjects with TRS were randomised to receive YKS, the remaining 64 received placebo, 3 of which did not complete the study. Authors found a significant decrease in the excitement/hostility factor of PANSS only (-1.65 ± 0.38 in YKS vs -0.38 ±0.36 in placebo, p = 0.018). No significant adverse effects were found with YKS.

Modabber et al. (2009)^16^ also studied allopurinol, however this was not included in the meta-analysis due to the absence of information for the baseline scores of participants. 17 subjects received allopurinol 300mg BD and placebo in a crossover design. No significant differences were observed between the two treatment periods.

Peselow et al. (1987)^17^ studied cholecystekinin octapeptide at two different doses (0.02mcg/kg and 0.04 mcg/kg) compared to placebo in 30 patients with schizophrenia. No consistent therapeutic effects were found for either dose.

Rahmanzadeh et al. (2016)^18^ studied bumetanide (1mg BD for 8 weeks), a diuretic which acts as a selective Na-K-Cl cotransporter 1 inhibitor, in 12 subjects compared to 12 subjects in the placebo group. The study looked at audioverbal hallucinations specifically, and found a significant reduction in the PANSS-P3 factor (reducing from 5.8 ±1.1 to 2.6 ± 1 in the bumetanide group vs 5.1 ±1.1 to 5 ±1 in the placebo group, p<0.001). Bumetanide was well-tolerated.

Sheitman et al. (2004)^19^ studied intravenous secretin, a neuropeptide which had previously shown beneficial behavioural effects in children with autistic spectrum disorder, in 11 subjects compared to 11 in the placebo group. No significant differences in symptom scores were found after controlling for baseline symptom severity, however more patients in the intervention group were rated as responders based on CGI scores. CGI ratings showed a significant improvement in the patients receiving secretin at week 3 (3.8 ± 0.4 in the secretin group vs 4.5 ± 0.5 in the placebo group).

1. **Review of intervention acceptibility**

*Antipsychotics*

As may be expected, patients receiving higher doses of antipsychotics generally experienced higher rates of extrapyramidal symptoms (EPS) and sedation in terms of absolute numbers, but there was only a trend towards a statistically higher rates in the high-dose groups reported in one study. This is with the exception of quetiapine, which showed similarly low rates of EPS in both groups, but did report a nonsignificantly higher mean weight gain in the high-dose group of 1.8lb versus 0.2lb. In a study investigating dose increment vs continuation of olanzapine vs risperidone, the rates of drop-outs in the increment group were over 10 times that of the continuation group (23.1% vs. 2%), with sedation accounting for the vast majority of these withdrawals.

In one study^20^, high-dose lurasidone led to greater memory-guided spatial errors on an eye movement task compared to the low-dose group, suggesting that cognitive dysfunction may emerge with higher doses. Another study also measured cognitive effects of high-dose lurasidone, finding that the standard-dose, but not high-dose, group showed improvement in both the Wisconsin Card Sorting Test and Digit Symbol Substitution Test, while the high-dose group showed significant deterioration in the Wisconsin Card Sorting Test.

*Glutamatergic interventions*

Adverse effects were not quantitatively described, but authors reported that both glycine and d-serine/d-cycloserine were well-tolerated throughout all included studies.

*Anticonvulsants*

No adverse effects were described with lamotrigine. With topiramate, symptoms of hypersalivation, aesthenia, memory disturbance and sedation were not statistically different between groups.

*Serotonergic interventions*

Ondansetron was found not just to be well-tolerated, but ameliorated adverse effects of co-administered antipsychotics. Antidepressants were generally well tolerated. Antidepressants were well-tolerated. Two studies reported extrapyramidal symptoms, and one study reported dry mouth and emesis in a minority of patients. However, none of these symptoms were statistically more frequent in the intervention group.

*Non-invasive stimulation*

Seven studies did not report on adverse effects. In those that did, no serious adverse events were reported. Headache was reported in 12 studies, and stimulation site effects, e.g. scalp burning, redness or itching were reported in nine; these were uniformly mild and resolved quickly after stimulation. Where statistical analysis was performed, none of these side effects were significantly more frequent in treatment or placebo arms.

*Psychotherapy*

Adverse effects related to psychotherapy were not reported.

*Famotidine*

Adverse effects were not reported in detail in either study, it was only mentioned in Meskanen et al. that there were no serious adverse events and none of the 30 participants withdrew from the study.

*Ginkgo biloba*

Patients receiving *Gingko biloba* + haloperidol showed fewer adverse effects than those receiving only haloperidol. Both studies report that the group receiving *Gingko biloba* scored significantly lower on the Treatment Emergent Symptom Scale items 1 (behavioural toxicity) and 3 (symptoms of the nervous system) than the placebo group.

*Sodium nitroprusside*

Sodium nitroprusside was generally well-tolerated, however 2 participants in one study^21^ experienced hypotension during infusion, likely related to sodium nitroprusside’s activity as a vasodilator.

1. **eFigure 4** – Breakdown of risk of bias scores in each domain of the Cochrane RoB tool v2
2. **Results of meta-regressions**

Baseline symptom severity was not a moderator of effect size in the positive (β = -0.001, p = 0.53), negative (β = 0.02, p = 0.27) or total (β = 0.001, p = 0.95) domains for NIS studies, and was not a moderator of effect size on positive symptoms for psychotherapy studies (β = -0.04, p = 0.26). Antipsychotic dose at baseline, reported in 13 of 26 NIS studies, was also not a moderator of effect size in the positive (β = -0.001, p = 0.24), negative (β = -0.001, p = 0.09) or total (β = -0.002, p = 0.10) domains. 44 of the studies included in the meta-analysis reported data on the proportion of patients taking clozapine, including 10 NIS studies. The proportion of patients taking clozapine was not a significant moderator of symptom improvement effect size for positive (β = -0.13, p = 0.91), negative (β = 0.22, p = 0.84) or total (β = 0.88, p = 0.47) domains. The proportion of the subject sample with a schizoaffective diagnosis similarly was not a moderator of symptom improvement effect size for positive (β = 0.61, p = 0.38), negative (β = -0.57, p = 0.55) or total (β = -0.47, p = 0.66) domains.

1. **Results of Wald-type tests**

*Impact of bias on effect size*

Risk-of-bias findings are summarised in *Table 1* and a detailed breakdown is available in *Supplementary eFigure 4*, created using the *robvis* tool^22^. Most studies were low- or medium-risk of bias, with psychotherapy and antipsychotic studies carrying the highest general risk.

Wald-type tests were not significant for the effect of risk of bias on positive (z = 0.21, p = 0.84), negative (z = -0.11, p = 0.92) or total (z = 0.94, p = 0.35) symptom improvement in NIS studies.

Wald-type tests were not significant for the effect of risk of bias on on positive (z = 0.39, p = 0.69) negative (z = 0.98, p = 0.33) or total (z = 0.31, p = 0.75) symptom improvement in psychotherapy studies.

Wald-type tests were not significant for the effect of risk of bias on positive (z = 0.13, p = 0.90), negative (z = -0.97, p = 0.33) or total (z = 0.16, p = 0.88) symptom improvement in glycine receptor modulator studies.

A sensitivity analysis excluding studies with high RoB was also performed. The effect of psychotherapy studies on positive symptoms became nonsignificant (g = -0.60 [-1.22, 0.02]).

*Impact of TRS definition on effect size*

Definitions of treatment-resistance varied between studies; for example some only required non-response to one trial of antipsychotic medication, where others required two or more trials. Wald-type tests were used to investigate whether there was a significant difference in effect size between studies using a lenient and strict definition of TRS, where there were ≥2 studies in both the lenient and strict definition categories; as such, this was performed for NIS, psychotherapy and antipsychotics in the three symptom domains.

Wald-type tests were not significant for the effect of TRS definition on positive (z = -0.80 p = 0.42), negative (z = -0.37, p = 0.71) or total (z = -0.67 p = 0.50) symptom improvement in psychotherapy studies.

Wald-type tests were not significant for the effect of TRS definition on negative (z = 0.11, p = 0.91) or total (z = 0.11, p = 0.91) symptom improvement in NIS studies.

Wald-type tests were not significant for the effect of TRS definition on positive (z = -1.1, p = 0.27), negative (z = -0.29, p = 0.78) or total (z = -0.79, p = 0.43) symptom improvement in antipsychotic studies.

Sensitivity analysis found that the significant effect of NIS on positive symptoms remained when studies using a lenient definition were excluded, although the magnitude of the effect was reduced (g = -0.26 [-0.49, 0.04], I^2^ = 48.6%).

1. **Results of sensitivity analyses excluding studies with significant conflicts of interest**

Most studies reported no conflict of interest and were funded by governmental or nonprofit funding sources. Many studies included authors reporting various honoraria or past funding from pharmaceutical or biotechnology companies. These were not assessed to be major conflicts of interest unless directly related to the study being reported. 12 studies included in the meta-analysis were assessed as having major conflicts of interest. These included studies sponsored by the manufacturer of the study drug^20,23,24^, one study where the study drug was supplied by its manufacturer^25^, one study where an author founded a company marketing the stimulation technique (although they did not use said company’s devices in the study)^26^ and 7 glycine receptor modulatory studies where one of the authors owned the intellectual property rights to use of d-serine in schizophrenia^6,7,27-31^.

Excluding two antipsychotic studies^20,23^ did not change the significance of results in the positive (g = 0.03 [-0.20, 0.27]), negative (g = 0.05 [-0.15, 0.27]) or total (g = 0.06 [-0.14, 0.26]) symptom domains.

Excluding three anticonvulsant studies^24,25^ did not change the significance of results in the positive (g = -0.40 [-1.16, 0.34]), negative (g = -0.16 [-1.15, 0.82]) or total (g = -0.53 [-0.98, 0.08]) symptom domains.

Excluding one non-invasive stimulation study^26^ did not change the significance of results in the positive (g = -0.44 [-1.68, 0.19]), negative (g = -0.09 [-0.31, 0.13]) or total (g = -0.14 [-0.40, 0.13]) symptom domains.

1. **GRADE assessment rationales for each outcome**

**Antipsychotic, positive:** LOW

- *Risk of bias:* **Very serious concerns** (-2)**.** The majority of studies had high risk of bias due to missing data, which may have been related to outcome.
- *Inconsistency:* **No serious concerns.** Heterogeneity was low.
- *Imprecision:* **No serious concerns.** Confidence intervals crossed thresholds for small effect size and the null effect line.
- *Indirectness:* **No serious concerns.** 3 of the 5 studies used TRS definitions meeting the TRRIP criteria.
- *Publication bias:* **Unlikely.** Although there were only 5 studies, funnel plots did not show asymmetry indicative of publication bias.
- *Other:* The fact that **different agents were used in most of the studies** led to downgrading the GRADE recommendations (-1).

**Antipsychotic, negative:** LOW

- *Risk of bias:* **Very serious concerns** (-2)**.** The majority of studies had high risk of bias due to missing data, which are likely to have been related to outcome.
- *Inconsistency:* **No serious concerns.** Heterogeneity was low.
- *Imprecision:* **No serious concerns.** Confidence intervals crossed the thresholds for small effect sizes and the null effect line only.
- *Indirectness:* **No serious concerns.** 3 of the 6 studies used TRS definitions meeting the TRRIP criteria.
- *Publication bias:* **Unlikely.** Although there were only 6 studies, funnel plots did not show asymmetry indicative of publication bias.
- *Other:* The fact that **different agents were used in most of the studies** led to downgrading the GRADE recommendations (-1).

**Antipsychotic, total:** VERY LOW

- *Risk of bias:* **Very serious concerns** (-2)**.** The majority of studies had high risk of bias due to missing data, which may have been related to outcome.
- *Inconsistency:* **No serious concerns.** Heterogeneity was generally low.
- *Imprecision:* **No serious concerns.** Confidence intervals crossed the null effect line only, suggesting relatively high precision estimate of no effect.
- *Indirectness:* **Serious concerns** (-1)**.** Only 3 of the 7 studies used TRS definitions meeting the TRRIP criteria.
- *Publication bias:* **Unlikely.** Funnel plots did not show asymmetry indicative of publication bias.
- *Other:* The fact that **different agents were used in most of the studies** led to downgrading the GRADE recommendations (-1).

**Ondansetron, positive:** VERY LOW

- *Risk of bias:* **Serious concerns** (-1)**.** While only 1 of 2 studies had a high RoB, although this was due to potentially selective reporting.
- *Inconsistency:* **Serious concerns** (-1). Heterogeneity was substantial.
- *Imprecision:* **Serious concerns** (-1). Confidence intervals crossed the medium and small effect size thresholds, and the null effect line.
- *Indirectness:* **No serious concerns**. 2 of the 3 studies used TRS definitions meeting the TRRIP criteria.
- *Publication bias:* Not assessed, as there were only 3 studies.
- *Other:* The **paucity of studies** and **small total sample size** led to the GRADE rating being downgraded (-3).

**Ondansetron, negative:** VERY LOW

- *Risk of bias:* **Very serious concerns** (-1)**.** While only 1 of 2 studies had a high RoB, although this was due to potentially selective reporting and was associated with a very large effect size.
- *Inconsistency:* **Serious concerns** (-1). Heterogeneity was substantial.
- *Imprecision:* **Very serious concerns** (-2). Confidence intervals crossed the large, medium and small effect size thresholds, the null effect line, and multiple thresholds which might suggest a deleterious effect on symptoms.
- *Indirectness:* **No serious concerns**. 2 of the 3 studies used TRS definitions meeting the TRRIP criteria.
- *Publication bias:* Not assessed, as there were only 2 studies.
- *Other:* The **paucity of studies** and **small total sample size** led to the GRADE rating being downgraded (-3).

**Antidepressants, positive**: VERY LOW

- *Risk of bias:* **No serious concerns.** Only 1 of the 3 studies had a high RoB, due to missing data.
- *Inconsistency:* **Serious concerns** (-1). Heterogeneity was substantial.
- *Imprecision:* **Very serious concerns** (-2). Confidence intervals crossed the large, medium and small effect size thresholds, the null effect line, and multiple thresholds which might suggest a deletirious effect on symptoms.
- *Indirectness:* **No serious concerns.** All studies used TRS definitions meeting the TRRIP criteria.
- *Publication bias:* Not assessed, as there were only 3 studies.
- *Other:* The **paucity of studies** led to the GRADE rating being downgraded (-1).

**Antidepressants, negative:** VERY LOW

- *Risk of bias:* **No serious concerns.** Only 1 of the 3 studies had a high RoB, due to missing data.
- *Inconsistency:* **Serious concerns** (-1). Heterogeneity was substantial.
- *Imprecision:* **Serious concerns** (-1)**.** While confidence intervals did not cross the null effect line, they were broad, crossing the large, medium and small effect size thresholds.
- *Indirectness:* **No serious concerns.** All studies used TRS definitions meeting the TRRIP criteria.
- *Publication bias:* Not assessed, as there were only 3 studies.
- *Other:* The **paucity of studies** and **small total sample size** led to the GRADE rating being downgraded (-3).

**Antidepressants, total:** LOW

- *Risk of bias:* **No serious concerns.** Only 1 of the 3 studies had a high RoB, due to missing data.
- *Inconsistency:* **No serious concerns.** Heterogeneity was 0.
- *Imprecision:* **No serious concerns**. Confidence intervals crossed the large and medium effect size thresholds only.
- *Indirectness:* **No serious concerns.** All studies used TRS definitions meeting the TRRIP criteria.
- *Publication bias:* Not assessed, as there were only 3 studies.
- *Other:* The **paucity of studies** and **small total sample size** led to the GRADE rating being downgraded (-3).

**Psychotherapy, positive:** LOW

- *Risk of bias:* **Very serious concerns** (-2)**.** 6 of the 9 studies had a high or moderate RoB, due to missing data, potentially selective reporting, and lack of participant blinding. Although lack of blinding did not always lead to a downgrading of RoB score as per Cochrane flowchart, it could affect results.
- *Inconsistency:* **Serious concerns** (-1)**.** Heterogeneity was substantial.
- *Imprecision:* **No serious concerns.** While confidence intervals crossed thresholds for large, moderate and small effect sizes, they did not cross the null effect line.
- *Indirectness:* **No serious concerns.** 6 of the 10 studies used TRS definitions meeting the TRRIP criteria.
- *Publication bias:* **Unlikely.** Funnel plots did not show asymmetry indicative of publication bias.

**Psychotherapy, negative:** LOW

- *Risk of bias:* **Serious concerns** (-1)**.** 3 of the 6 studies had a high or moderate RoB, due to missing data, and all studies lacked participant blinding. Although lack of blinding did not always lead to a downgrading of RoB score as per Cochrane flowchart, it could affect results.
- *Inconsistency:* **Serious concerns** (-1)**.** Heterogeneity was substantial.
- *Imprecision:* **Serious concerns** (-1)**.** Confidence intervals crossed thresholds for small effect sizes, and the null effect line.
- *Indirectness:* **No serious concerns.** 4 of 8 studies used TRS definitions meeting the TRRIP criteria.
- *Publication bias:* **Unlikely.** Funnel plots did not show asymmetry indicative of publication bias.

**Psychotherapy, total:** LOW

- *Risk of bias:* **Serious concerns** (-1)**.** 3 of the 6 studies had a high or moderate RoB, due to missing data, and lack of participant blinding. Although lack of blinding did not always lead to a downgrading of RoB score as per Cochrane flowchart, it could affect results.
- *Inconsistency:* **Serious concerns** (-1)**.** Heterogeneity was substantial.
- *Imprecision:* **Serious concerns** (-1)**.** Confidence intervals crossed thresholds for large, moderate and small effect sizes, and the null effect line.
- *Indirectness:* **No serious concerns.** 5 of 8 studies used TRS definitions meeting the TRRIP criteria.
- *Publication bias:* **Unlikely.** Funnel plots did not show asymmetry indicative of publication bias.

**Non-invasive stimulation, positive:** LOW

- *Risk of bias:* **No serious concerns.**  11 of 26 studies had high or moderate RoB, due to missing data, although often few participants were missing, and due to the nature of the intervention and short duration of treatment, missingness was unlikely to be related to outcome.
- *Inconsistency:* **Serious concerns** (-1)**.** Heterogeneity was substantial.
- *Imprecision:* **No serious concerns**. Confidence intervals crossed the thresholds for moderate and small effect sizes only.
- *Indirectness:* **No serious concerns**. 19 of 26 studies used TRS definitions meeting the TRRIP criteria.
- *Publication bias:* **Strongly suspected** (-2)**.** Funnel plots and Egger’s tests showed evidence of publication bias.

**Non-invasive stimulation, negative:** VERY LOW

- *Risk of bias:* **No serious concerns.**  7 of 12 studies had high or moderate RoB, due to missing data, although often few participants were missing, and due to the nature of the intervention and short duration of treatment, missingness was unlikely to be related to outcome.
- *Inconsistency:* **Serious concerns** (-1)**.** Heterogeneity was substantial.
- *Imprecision:* **No serious concerns**. Confidence intervals crossed the thresholds for small effect size and the null effect line only.
- *Indirectness:* **No serious concerns**. 8 of 12 studies used TRS definitions meeting the TRRIP criteria.
- *Publication bias:* **Strongly suspected** (-2)**.** Funnel plots and Egger’s tests showed evidence of publication bias.
- *Other:* **Likely reporting biases** led to downgrading the GRADE rating (-1). Since many studies focused on improving auditory hallucinations as a primary outcome, negative symptom changes were less likely to be reported.

**Non-invasive stimulation, total:** VERY LOW

- *Risk of bias:* **No serious concerns.**  7 of 12 studies had high or moderate RoB, due to missing data, although often few participants were missing, and due to the nature of the intervention and short duration of treatment, missingness was unlikely to be related to outcome.
- *Inconsistency:* **Serious concerns** (-1)**.** Heterogeneity was substantial.
- *Imprecision:* **No serious concerns**. Confidence intervals crossed the thresholds for small effect size and the null effect line only.
- *Indirectness:* **No serious concerns**. 8 of 12 studies used TRS definitions meeting the TRRIP criteria.
- *Publication bias:* **Strongly suspected** (-2)**.** Funnel plots and Egger’s tests showed evidence of publication bias.
- *Other:* **Likely reporting biases** led to downgrading the GRADE rating (-1). Since many studies focused on improving auditory hallucinations as a primary outcome, total symptom changes were less likely to be reported.

**GMS agonist, positive:** LOW

- *Risk of bias:* **Serious concerns** (-1)**.** 6 of 9 studies had high or moderate RoB, due to missing data, which could feasibly have been related to outcome.
- *Inconsistency:* **No serious concerns.** Heterogeneity was minimal.
- *Imprecision:* **No serious concerns**. Confidence intervals crossed the thresholds for large and moderate effect sizes only.
- *Indirectness:* **No serious concerns**. All studies used TRS definitions meeting the TRRIP criteria.
- *Publication bias:* **Likely** (-1)**.** Although there were only 9 studies, funnel plots showed asymmetry which may be indicative of publication bias.
- *Other factors:* **Consistently large effect sizes** led to upgrading the GRADE rating (+1). However, the fact that **studies were small** and **the majority of studies were from the same research group** led to downgrading the GRADE rating (-2).

**Glutamatergic, negative:** LOW

- *Risk of bias:* **Serious concerns** (-1)**.** 5 of 8 studies had high or moderate RoB, due to missing data, which could feasibly have been related to outcome.
- *Inconsistency:* **No serious concerns.** Heterogeneity was low.
- *Imprecision:* **No serious concerns**. Confidence intervals did not cross any effect size threshold.
- *Indirectness:* **No serious concerns**. All studies used TRS definitions meeting the TRRIP criteria.
- *Publication bias:* **Likely** (-1)**.** Although there were only 8 studies, funnel plots showed asymmetry which may be indicative of publication bias.
- *Other factors:* **Consistently large effect sizes** led to upgrading the GRADE rating (+1). However, the fact that **studies were small** and **the majority of studies were from the same research group** led to downgrading the GRADE rating (-2).

**Glutamatergic, total:** VERY LOW

- *Risk of bias:* **Serious concerns** (-1)**.** 6 of 9 studies had high or moderate RoB, due to missing data, which could feasibly have been related to outcome.
- *Inconsistency:* **Serious concerns** (-1)**.** Heterogeneity was substantial.
- *Imprecision:* **No serious concerns**. Confidence intervals did not cross any effect size threshold.
- *Indirectness:* **No serious concerns**. All studies used TRS definitions meeting the TRRIP criteria.
- *Publication bias:* **Likely** (-1)**.** Although there were only 9 studies, funnel plots showed asymmetry which may be indicative of publication bias.
- *Other factors:* **Large effect sizes** led to upgrading the GRADE rating (+1). However, the fact that **studies were small** and **the majority of studies were from the same research group** led to downgrading the GRADE rating (-2).

***Ginkgo biloba*, positive:** VERY LOW

- *Risk of bias:* **Serious concerns** (-1)**.**  1 of the 2 studies had high RoB as it did not report on how many patients withdrew from the study, which could feasibly have related to lack of efficacy.
- *Inconsistency:* **No serious concerns.** Heterogeneity was 0%.
- *Imprecision:* **Serious concerns** (-1). Confidence intervals were broad and crossed the thresholds for moderate and small effect sizes.
- *Indirectness:* **No serious concerns**. Both studies used TRS definitions meeting the TRRIP criteria.
- *Publication bias:* **Not assessed**. Only two studies were included.
- *Other factors*: The **paucity of studies** led to downgrading the GRADE rating (-2).

***Ginkgo biloba*, negative:** LOW

- *Risk of bias:* **Serious concerns** (-1)**.**  1 of the 2 studies had high RoB as it did not report on how many patients withdrew from the study, which could feasibly have related to lack of efficacy.
- *Inconsistency:* **No serious concerns.** Heterogeneity was 0%.
- *Imprecision:* **No serious concerns**. Confidence intervals were crossed the thresholds for small effect size and the null effect line only.
- *Indirectness:* **No serious concerns**. Both studies used TRS definitions meeting the TRRIP criteria.
- *Publication bias:* **Not assessed**. Only two studies were included.
- *Other factors*: The **paucity of studies** led to downgrading the GRADE rating (-2).

**Famotidine, total:** VERY LOW

- *Risk of bias:* **Very serious concerns** (-2)**.**  1 of the 2 studies had high RoB due to potentially selective reporting.
- *Inconsistency:* **No serious concerns.** Heterogeneity was minimal.
- *Imprecision:* **No serious concerns**. Confidence intervals crossed the thresholds for large effect size only.
- *Indirectness:* **No serious concerns**. Both studies used TRS definitions meeting the TRRIP criteria.
- *Publication bias:* **Not assessed**. Only two studies were included.
- *Other factors*: The **paucity of studies** led to downgrading the GRADE rating (-2).

**Mood stabilisers, positive:** LOW

- *Risk of bias:* **No serious concerns.**  All studies had low RoB.
- *Inconsistency:* **Serious concerns** (-1)**.** Heterogeneity was considerable.
- *Imprecision:* **Serious concerns** (-1). Confidence intervals crossed the thresholds for small effect sizes only both sides of the null effect line.
- *Indirectness:* **Very serious concerns** (-2). Only 1 study used TRS definition meeting the TRRIP criteria.
- *Publication bias:* **Unlikely.** Although only 4 studies were included, funnel plots did not show asymmetry indicative of publication bias.

**Mood stabilisers, negative:** LOW

- *Risk of bias:* **No serious concerns.**  All studies had low RoB.
- *Inconsistency:* **Serious concerns** (-1)**.** Heterogeneity was moderate.
- *Imprecision:* **No serious concerns**. Confidence intervals crossed the thresholds for the null effect line, and the small effect size threshold indicating deletirious effects on negative symptoms only.
- *Indirectness:* **Very serious concerns** (-2). Only 1 study used TRS definition meeting the TRRIP criteria.
- *Publication bias:* **Unlikely.** Although only 5 studies were included, funnel plots did not show asymmetry indicative of publication bias.

**Mood stabilisers, total:** LOW

- *Risk of bias:* **No serious concerns.**  All studies had low RoB.
- *Inconsistency:* **Serious concerns** (-1)**.** Heterogeneity was moderate.
- *Imprecision:* **No serious concerns**. Confidence intervals crossed the thresholds for the small effect size and null effect line only.
- *Indirectness:* **Very serious concerns** (-2). Only 1 study used TRS definition meeting the TRRIP criteria.
- *Publication bias:* **Unlikely.** Although only 5 studies were included, funnel plots did not show asymmetry indicative of publication bias.

**Sodium nitroprusside, positive:** VERY LOW

- *Risk of bias:* **Serious concerns** (-1)**.**  Both studies had some risk of bias, one of which was due to missing data which could have been related to outcome.
- *Inconsistency:* **No serious concerns.** Heterogeneity was minimal.
- *Imprecision:* **Serious concerns** (-1). Confidence intervals crossed several thresholds including the null effect line.
- *Indirectness:* **Serious concerns** (-1). Only one of the two studies used a TRS definition meeting the TRRIP criteria.
- *Publication bias:* **Not assessed**. Only two studies were included.
- *Other factors*: **The** **paucity of studies** led to downgrading the GRADE rating (-2).

**Sodium nitroprusside, negative:** VERY LOW

- *Risk of bias:* **Serious concerns** (-1)**.**  Both studies had some risk of bias, one of which was due to missing data which could have been related to outcome.
- *Inconsistency:* **No serious concerns.** Heterogeneity was minimal.
- *Imprecision:* **No serious concerns**. Confidence intervals crossed the thresholds for large effect size only.
- *Indirectness:* **Serious concerns** (-1). Only one of the two studies used a TRS definition meeting the TRRIP criteria.
- *Publication bias:* **Not assessed**. Only two studies were included.
- *Other factors*: The **paucity of studies** led to downgrading the GRADE rating (-2).

**Sodium nitroprusside, total:** VERY LOW

- *Risk of bias:* **Serious concerns** (-1)**.**  Both studies had some risk of bias, one of which was due to missing data which could have been related to outcome.
- *Inconsistency:* **No serious concerns.** Heterogeneity was minimal.
- *Imprecision:* **No serious concerns**. Confidence intervals crossed the thresholds for large effect size only.
- *Indirectness:* **Serious concerns** (-1). Only one of the two studies used a TRS definition meeting the TRRIP criteria.
- *Publication bias:* **Not assessed**. Only two studies were included.
- *Other factors*: The **paucity of studies** led to downgrading the GRADE rating (-2).

REFERENCES FOR SUPPLEMENTAL MATERIAL

1. Siskind D, McCartney L, Goldschlager R, Kisely S. Clozapine v. first-and second-generation antipsychotics in treatment-refractory schizophrenia: systematic review and meta-analysis. *The British Journal of Psychiatry*. 2016;209(5):385-392.

2. Samara MT, Dold M, Gianatsi M, et al. Efficacy, Acceptability, and Tolerability of Antipsychotics in Treatment-Resistant Schizophrenia: A Network Meta-analysis. *JAMA Psychiatry*. Mar 2016;73(3):199-210. doi:10.1001/jamapsychiatry.2015.2955

3. Chakos M, Lieberman J, Hoffman E, Bradford D, Sheitman B. Effectiveness of second-generation antipsychotics in patients with treatment-resistant schizophrenia: a review and meta-analysis of randomized trials. *American Journal of Psychiatry*. 2001;158(4):518-526.

4. Dong S, Schneider-Thoma J, Bighelli I, et al. A network meta-analysis of efficacy, acceptability, and tolerability of antipsychotics in treatment-resistant schizophrenia. *European Archives of Psychiatry and Clinical Neuroscience*. 2023:1-12.

5. Higgins JP TJ, Chandler J, Cumpston M, Li T, Page MJ, Welch VA, editor(s). *Cochrane Handbook for Systematic Reviews of Interventions Version 6.2* Cochrane; 2021. Accessed 20/June/2023. training.cochrane.org/handbook.

6. Heresco-Levy U, Javitt DC, Ermilov M, Mordel C, Horowitz A, Kelly D. Double-blind, placebo-controlled, crossover trial of glycine adjuvant therapy for treatment-resistant schizophrenia. *The British Journal of Psychiatry*. 1996;169(5):610-617.

7. Heresco-Levy U, Ermilov M, Lichtenberg P, Bar G, Javitt DC. High-dose glycine added to olanzapine and risperidone for the treatment of schizophrenia. *Biological psychiatry*. 2004;55(2):165-171.

8. Kremer I, Vass A, Gorelik I, et al. Placebo-controlled trial of lamotrigine added to conventional and atypical antipsychotics in schizophrenia. *Biological psychiatry*. 2004;56(6):441-446.

9. Brunelin J, Mondino M, Gassab L, et al. Examining transcranial direct-current stimulation (tDCS) as a treatment for hallucinations in schizophrenia. *American Journal of Psychiatry*. 2012;169(7):719-724.

10. Brunstein MG, Ghisolfi ES, Ramos FL, Lara DR. A clinical trial of adjuvant allopurinol therapy for moderately refractory schizophrenia. *Journal of Clinical Psychiatry*. 2005;66(2):213-219.

11. Goswami U, Kumar U, Singh B. Efficacy of electroconvulsive therapy in treatment resistant schizophreinia: a double-blind study. *Indian journal of psychiatry*. 2003;45(1):26.

12. Kulkarni J, Gavrilidis E, Wang W, et al. Estradiol for treatment-resistant schizophrenia: a large-scale randomized-controlled trial in women of child-bearing age. *Molecular psychiatry*. 2015;20(6):695-702.

13. Marco EJ, Wolkowitz OM, Vinogradov S, Poole JH, Lichtmacher J, Reus VI. Double-blind antiglucocorticoid treatment in schizophrenia and schizoaffective disorder: a pilot study. *World J Biol Psychiatry*. Jul 2002;3(3):156-61. doi:10.3109/15622970209150617

14. Mishra A, Reeta KH, Sarangi SC, Maiti R, Sood M. Effect of add-on alpha lipoic acid on psychopathology in patients with treatment-resistant schizophrenia: a pilot randomized double-blind placebo-controlled trial. *Psychopharmacology*. 2022/11/01 2022;239(11):3525-3535. doi:10.1007/s00213-022-06225-2

15. Miyaoka T, Furuya M, Horiguchi J, et al. Efficacy and safety of yokukansan in treatment-resistant schizophrenia: a randomized, double-blind, placebo-controlled trial (a Positive and Negative Syndrome Scale, five-factor analysis). *Psychopharmacology*. 2015/01/01 2015;232(1):155-164. doi:10.1007/s00213-014-3645-8

16. Modabber A, Najmedin ST. Allopurinol as an adjuvant therapy for refractory schizophrenia. *Pakistan Journal of Medical Sciences*. 2009;25(4):591-596.

17. Peselow E, Angrist B, Sudilovsky A. Double blind controlled trials of cholecystokinin octapeptide in neuroleptic-refractory schizophrenia. *Psychopharmacology*. 1987;91(1):80-84. doi:<https://dx.doi.org/10.1007/BF00690931>

18. Rahmanzadeh R, Eftekhari S, Shahbazi A, et al. Effect of bumetanide, a selective NKCC1 inhibitor, on hallucinations of schizophrenic patients; a double-blind randomized clinical trial. *Schizophr Res*. Jun 2017;184:145-146. doi:10.1016/j.schres.2016.12.002

19. Sheitman BB, Knable MB, Jarskog LF, et al. Secretin for refractory schizophrenia. *Schizophr Res*. Feb 1 2004;66(2-3):177-81. doi:10.1016/s0920-9964(03)00068-9

20. Karpouzian-Rogers T, Stocks J, Meltzer HY, Reilly JL. The effect of high vs. low dose lurasidone on eye movement biomarkers of prefrontal abilities in treatment-resistant schizophrenia. *Schizophr Res*. Jan 2020;215:314-321. doi:10.1016/j.schres.2019.10.008

21. Brown HE, Freudenreich O, Fan X, et al. Efficacy and Tolerability of Adjunctive Intravenous Sodium Nitroprusside Treatment for Outpatients With Schizophrenia: A Randomized Clinical Trial. *JAMA Psychiatry*. Jul 1 2019;76(7):691-699. doi:10.1001/jamapsychiatry.2019.0151

22. McGuinness LA, Higgins JPT. Risk-of-bias VISualization (robvis): An R package and Shiny web app for visualizing risk-of-bias assessments. *Research Synthesis Methods*. 2020/04/26 2020;n/a(n/a)doi:10.1002/jrsm.1411

23. Meltzer HY, Share DB, Jayathilake K, Salomon RM, Lee MA. Lurasidone Improves Psychopathology and Cognition in Treatment-Resistant Schizophrenia. *J Clin Psychopharmacol*. May-Jun 2020;40(3):240-249. doi:10.1097/jcp.0000000000001205

24. Goff DC, Keefe R, Citrome L, et al. Lamotrigine as add-on therapy in schizophrenia: results of 2 placebo-controlled trials. *J Clin Psychopharmacol*. Dec 2007;27(6):582-9. doi:10.1097/jcp.0b013e31815abf34

25. Wilson WH. Addition of lithium to haloperidol in non-affective, antipsychotic non-responsive schizophrenia: a double blind, placebo controlled, parallel design clinical trial. *Psychopharmacology (Berl)*. 1993;111(3):359-66. doi:10.1007/bf02244953

26. Mellin JM, Alagapan S, Lustenberger C, et al. Randomized trial of transcranial alternating current stimulation for treatment of auditory hallucinations in schizophrenia. *European Psychiatry*. 2018;51:25-33.

27. Javitt DC, Silipo G, Cienfuegos A, et al. Adjunctive high-dose glycine in the treatment of schizophrenia. *International Journal of Neuropsychopharmacology*. 2001;4(4):385-391.

28. Heresco-Levy U, Javitt DC, Ebstein R, et al. D-serine efficacy as add-on pharmacotherapy to risperidone and olanzapine for treatment-refractory schizophrenia. *Biological psychiatry*. 2005;57(6):577-585.

29. Heresco-Levy U, Javitt DC, Ermilov M, Silipo G, Shimoni J. Double-blind, placebo-controlled, crossover trial of D-cycloserine adjuvant therapy for treatment-resistant schizophrenia. *International Journal of Neuropsychopharmacology*. 1998;1(2):131-135.

30. Heresco-Levy U, Javitt DC, Ermilov M, Mordel C, Silipo G, Lichtenstein M. Efficacy of high-dose glycine in the treatment of enduring negative symptoms of schizophrenia. *Archives of general psychiatry*. 1999;56(1):29-36.

31. Heresco-Levy U, Ermilov M, Shimoni J, Shapira B, Silipo G, Javitt DC. Placebo-controlled trial of D-cycloserine added to conventional neuroleptics, olanzapine, or risperidone in schizophrenia. *American Journal of Psychiatry*. 2002;159(3):480-482.
